# Rev1 contributes to proper mitochondrial function via the PARP-NAD^+^-SIRT1-PGC1α axis

**DOI:** 10.1038/s41598-017-12662-3

**Published:** 2017-10-02

**Authors:** Nima Borhan Fakouri, Jon Ambæk Durhuus, Christine Elisabeth Regnell, Maria Angleys, Claus Desler, Md Mahdi Hasan-Olive, Ana Martín-Pardillos, Anastasia Tsaalbi-Shtylik, Kirsten Thomsen, Martin Lauritzen, Vilhelm A. Bohr, Niels de Wind, Linda Hildegard Bergersen, Lene Juel Rasmussen

**Affiliations:** 1 Center for Healthy Aging, Department of Cellular and Molecular Medicine, University of Copenhagen, Copenhagen, Denmark; 2 Department of Oral Biology, University of Oslo, Oslo, Norway; 3 Leiden University Medical Center, Leiden, Netherlands; 4 Center for Healthy Aging, Department of Neuroscience and Pharmacology, University of Copenhagen, Copenhagen, Denmark; 5 Department of Clinical Neurophysiology, Rigshospitalet, 2600 Glostrup, Denmark; 6 National Institute on Aging, NIH, Baltimore, USA

## Abstract

Nucleic acids, which constitute the genetic material of all organisms, are continuously exposed to endogenous and exogenous damaging agents, representing a significant challenge to genome stability and genome integrity over the life of a cell or organism. Unrepaired DNA lesions, such as single- and double-stranded DNA breaks (SSBs and DSBs), and single-stranded gaps can block progression of the DNA replication fork, causing replicative stress and/or cell cycle arrest. However, translesion synthesis (TLS) DNA polymerases, such as Rev1, have the ability to bypass some DNA lesions, which can circumvent the process leading to replication fork arrest and minimize replicative stress. Here, we show that Rev1-deficiency in mouse embryo fibroblasts or mouse liver tissue is associated with replicative stress and mitochondrial dysfunction. In addition, Rev1-deficiency is associated with high poly(ADP) ribose polymerase 1 (PARP1) activity, low endogenous NAD^+^, low expression of SIRT1 and PGC1α and low adenosine monophosphate (AMP)-activated kinase (AMPK) activity. We conclude that replication stress via Rev1-deficiency contributes to metabolic stress caused by compromized mitochondrial function via the PARP-NAD^+^-SIRT1-PGC1α axis.

## Introduction

DNA is continuously challenged by endogenous DNA damaging agents, including reactive oxygen species (ROS) and reactive aldehydes, that are present in normal cells under normal physiological conditions. It has been estimated that more than 10,000 oxidative DNA lesions arise spontaneously per mammalian cell per day^1,2^. Many types of endogenous DNA damage are quickly removed by base excision repair (BER), nucleotide excision repair (NER), mismatch repair (MMR) or double strand break repair (DSBR)^3^ pathways. However, DNA lesions that persist due to inefficient repair can be mutagenic, and/or can inhibit DNA replication/transcription, trigger apoptosis, cell cycle arrest or cellular senescence, or activate the DNA damage response (DDR) pathway^4^. Recent studies demonstrate that constitutive DDR consumes a large amount of energy in the form of adenosine triphosphate (ATP) and/or nicotinamide adenine dinucleotide (NAD^+^), such that chronic replicative stress is frequently associated with metabolic stress caused by energy imbalance or poor energy homeostasis^5–7^. Constitutive activation of DDR also creates a vicious cycle, as it can increase the abundance of ROS, which in turn generates more oxidative DNA lesions^8^. A key enzyme in DDR is poly(ADP) ribose polymerase 1 (PARP1), whose high consumption of intracellular NAD^+^ can contribute to depletion of this important metabolite and lead to mitochondrial dysfunction^9–11^.

When a cell’s capacity to repair DNA lesions is exceeded, persistent DNA lesions arise, some of which block the progression of DNA replication forks. Typically, a cell responds by activating additional origins of replication, or by activating translesion synthesis (TLS)^4^ DNA polymerases, such as Rev1. Rev1 belongs to the Y-family of DNA polymerases, enzymes that carryout post-replication gap-filling DNA synthesis using a lesion-containing DNA strand as a template for the TLS reaction^12,13^. Rev1 also recruits other TLS polymerases to sites of DNA damage^14–16^. TLS gene mutations and sequence variants are involved in many human neurological disorders as well as cancer^17–20^.

In *Saccharomyces cerevisiae*, The first 148 amino acids of Rev1 are sufficient to direct the localization of GFP to the mitochondria of yeast and Rev1 is thought to play a role in mutagenesis in that organelle^21^. In Rev1-deficient mouse embryo fibroblasts (*Rev1*^−/−^ MEFs), ssDNA gaps accumulate during DNA replication^13^ and over time, proliferating tissues and stem cells are depleted in *Rev1*^−/−^ mice relative to wild type (WT) controls^22^. *Rev1*^−/−^ mice also have a growth defect^13,23^. To date, defects in Rev1 have not been linked to mitochondrial dysfunction in mammalian cells.

This study provides evidence that Rev1 is required for normal mitochondrial function and energy homeostasis in mouse cells. Furthermore, data presented here suggest that *Rev1*^−/−^ MEFs express increased levels of constitutively activated PARP1, reduced levels of endogenous NAD^+^, reduced expression of SIRT1 and PGC1α, reduced adenosine monophosphate (AMP)-activated kinase (AMPK) activity, and reduced autophagy and mitophagy. We conclude that replication stress caused by Rev1-deficiency contributes to cellular and organismal metabolic stress via mitochondrial dysfunction involving the PARP-NAD^+^-SIRT1-PGC1α axis^7^.

## Results

In *S. cerevisiae* Rev1 localizes to the mitochondria, where it is thought to participate in replication and mutagenesis of the mitochondrial genome (mtDNA)^21,24,25^. Our past studies also suggest that *S. cerevisiae* Rev1, as well as Rev3, and Rev7, plays a role in mitochondrial-mediated mutagenesis^26^. The goal of the present study was to explore if Rev1 plays a role in mitochondrial function in mammalian cells.

### Mitochondrial function is impaired in *Rev1*^−/−^ cells

**Figure 1:**
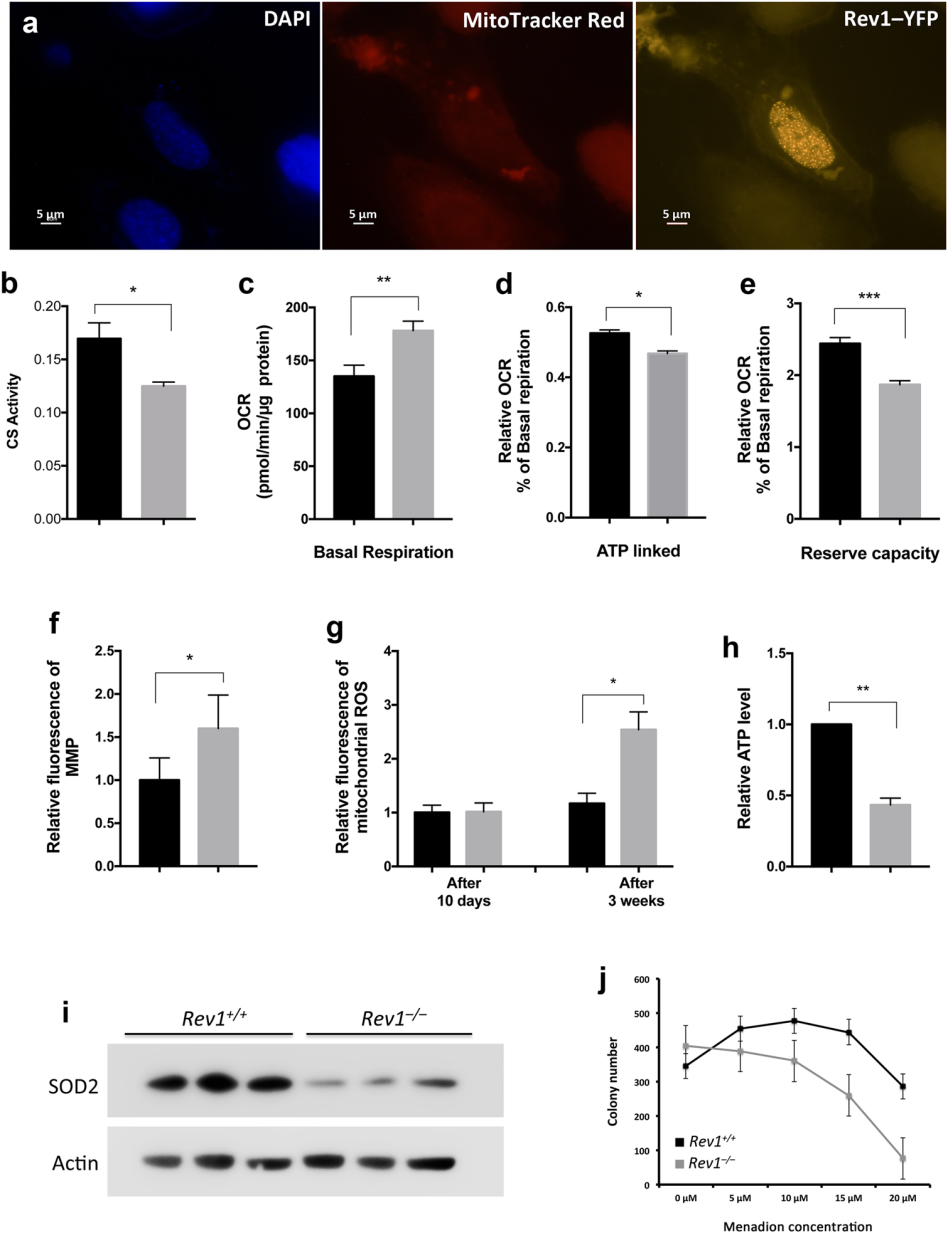
Mitochondrial function is impaired in *Rev1*^−/−^ Cells. (**a**) Fluorescent microscopy analysis of subcellular localization of Rev1 protein. *Rev1*^−/−^ MEFs transfected with plasmid containing Rev1-YFP fusion protein. MitoTracker Red and DAPI were used to visualize mitochondria and nucleus respectively (Size bar: 5 μM). (**b**) CS activity in the liver of 5 months old WT and *Rev1*^−/−^ mice. (**c**–**e)** Basal and ATP linked respiration and reserve capacity in WT and *Rev1*^−/−^ MEF cells (n = 5). Basal respiration was calculated and normalized to the protein content (t = 4.808; df = 4). ATP linked respiration and reserve capacity were measured upon the injection of oligomycine (0.3 μM) and FCCP (1.5 μM) respectively. ATP linked respiration (t = 3.875; df = 4) and reserve capacity (t = 11.68; df = 4) are represented as relative decrease and increase in OCR, respectively, compared to basal respiration. (**f**,**g)** Flow cytometry analysis of MMP and mitochondrial ROS in WT and *Rev1*^−/−^ MEF cells. The MMP was measured 3 weeks after initiation of cell culture (n = 4; t = 3.29; df = 3). The mitochondrial ROS was measured after 10 days and again after 3 weeks (n = 3; t = 9.777; df = 2). (**h)** The relative ATP level in WT and *Rev1*^−/−^ MEFs after 3 weeks (n = 3; t = 11.7 df = 2). (**i)** Expression of SOD2 (mnSOD) in WT and *Rev1*^−/−^ MEFs (n = 3). (**j)** Colony forming assay, cells treated with increasing concentrations of menadione. Colonies were stained with crystal violate and counted after one week of growth. n = sample number; t = t value; df = degree of freedom; *p < 0.05. **p < 0.01. ***p < 0.001. ****p < 0.0001. Data presented are mean ± M.S.E. Dark bars: WT and gray bars: *Rev1*^−/−^.

While the subcellular localization of murine Rev1 has not been determined, several reports indicate that human Rev1 is predominantly a nuclear protein^27,28^. Results presented in Fig. 1a suggest that murine Rev1 localizes predominantly to the nucleus. However, it might still affect mitochondrial functions.

Mitochondrial parameters were measured in primary liver cells from 5 month old WT and *Rev1*^−/−^ mice (Figure S1) and in WT and *Rev1*^−/−^ MEFs (Figs. 1c–e). Standard measures of electron transport chain (ETC) activity and oxidative phosphorylation (OXPHOS) were performed in an extracellular flux analyzer (Seahorse); these parameters were similar in WT and *Rev1*^−/−^ liver tissue (Figures S1a, S1b); however, citrate synthase (CS) activity was lower in *Rev1*^−/−^ liver tissue compared to WT (Fig. 1b). In contrast the basal respiration rate was approximately 30% higher in *Rev1*^−/−^ MEFs than in WT MEFs (Fig. 1c). In MEFs treated with oligomycin to inhibit ATP synthase (complex V), respiration was 52% of basal respiration in WT and 46% of basal respiration in *Rev1*^−/−^ cells (Fig. 1d). One interpretation is that ATP production is increased in *Rev1*^−/−^ MEFs compared to WT MEFs^29^. The results also showed that reserve capacity was approximately 25% lower in *Rev1*^−/−^MEFs than in WT MEFs (Fig. 1e). This suggests that loss of Rev1 may impair mitochondrial functions^29^. A constant increase in mitochondrial activity associates with increased mitochondrial membrane potential (MMP) and ROS production^8^. Membrane potential and ROS were elevated in *Rev1*^−/−^ MEFs (Fig. 1f,g, S6), although the level of ROS increased gradually over 3 weeks in culture. The latter increase was not observed in WT MEFs (Fig. 1g).

Cellular stress and increased demand for ATP can lead to increases in respiration and MMP^30,31^. To assess this, ATP abundance was measured in WT and *Rev1*^−/−^ MEFs (Fig. 1h). The results showed that total ATP was approximately 2-fold lower in *Rev1*^−/−^ MEFs than in WT MEFs. This could reflect either decreased synthesis and/or increased consumption of ATP.

### The expression of SOD2 is decreased in Rev1^−/−^ MEFs

Superoxide dismutase 2 (SOD2) is a mitochondrial matrix protein that converts highly reactive superoxide (O_2•_^−^) to less reactive hydrogen peroxide (H_2_O_2_), thereby protecting proteins, lipids, and DNA from oxidative damage^8,32,33^. Interestingly, SOD2 protein expression was decreased in *Rev1*^−/−^ MEFs compared to WT MEFs (Fig. 1i). This could contribute to the increased abundance of ROS in the mitochondria of *Rev1*^−/−^ cells^8,34^. Decreased SOD2 would also be expected to increase sensitivity of *Rev1*^−/−^ cells to oxidative stress. Menadione is a quinone, which is metabolized and reduced to semi-quinone by flavoprotein within the mitochondrial inner membrane. In the presence of molecular oxygen, it enters the redox cycle to generate ROS such as superoxide anion radicals, H2O2, and Hydroxyl radical, which can induce oxidative stress in cells (Figure S2)^34,35^. Consistent with this prediction, *Rev1*^−/−^ MEFs were more sensitive to menadione (5 to 20 μM) than WT MEFs (Fig. 1j), as measured by survival in a colony formation assay. The results also show that lower concentrations of menadione increase survival in WT MEFs, but not in *Rev1-*deficient MEFs (Fig. 1j).

### Mitochondrial OXPHOS proteins are more abundant in Rev1-deficient cells

**Figure 2:**
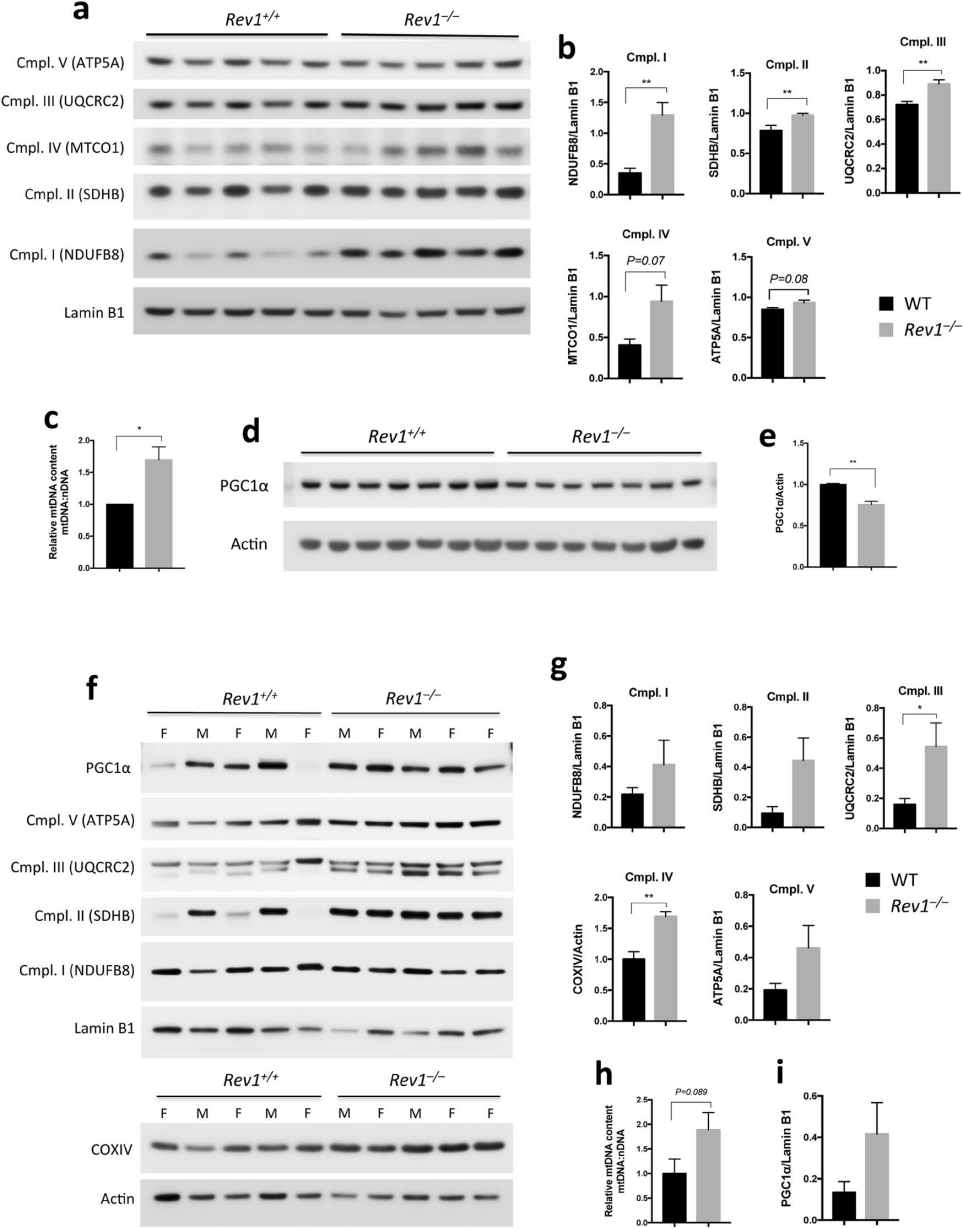
Mitochondrial content and biogenesis. (**a,b,f,g)** Immunoblot analysis of the subunits of mitochondrial complexes in MEF cells (n = 5; df = 4) **(a**,**b)** and in liver (n = 5; df = 8) **(f**,**g)**. (**b**,**g)** Quantifications of Cmpl. I-V protein levels from **(A)** MEFs and **(f)** liver, respectively. Cmpl. I (NDUFB8, NADH dehydrogenase 1 beta subcomplex subunit 8) (MEFs: t = 4.981; Liver: t = 1.17); Cmpl. II: SDHB (Succinate dehydrogenase iron-sulfur subunit) (MEFs: t = 3.685; Liver: t: 2.23); Cmpl. III: UQCRC2 (Cytochrome b-c1 complex subunit 2) (MEFs: t = 5.411; Liver: 2.383); Cmpl. IV: MTCO1 (Cytochrome c oxidase subunit 1) (MEFs: t = 2.378); COXIV (Cytochrome c oxidase subunit 4) (Liver: t = 4.79); Cmpl. V: ATP5A (ATP synthase subunit alpha) (MEFs: t = 2.302; Liver: t = 1.806). (**c**,**h)** Analysis of mtDNA content number by qPCR in MEF cells (n = 4; t = 3.544; df = 3) **(c)** and liver (n = 5; t = 1.944; df = 8) **(h)**. The mtDNA content was calculated as the mtDNA/nDNA ratio using primers specific for the mitochondrial TrnL1and nuclear β-2-microglobulin (β2M) genes. (**d)** Immunoblot analysis of PGC1α in MEFs (n = 7; t = 5.764; df = 6). (**e)** Quantification of PGC1α protein levels from **(d)** MEFs. (**i)** Quantification of PGC1α protein level in the liver of WT and *Rev1*^−/−^ 5 months old mice (t = 1.761). n = sample number; t = t value; df = degree of freedom; *p < 0.05. **p < 0.01. ***p < 0.001. ****p < 0.0001. Data presented are mean ± M.S.E.

We investigated the mitochondrial content by analyzing the abundance of selected OXPHOS subunits and mtDNA content in WT and *Rev1*^−/−^ liver tissue and MEFs. The results showed that complex I, II and III proteins are more abundant and mtDNA content is higher in *Rev1*^−/−^ MEFs than in WT MEFs (Fig. 2a–c). In liver cells from female mice, complex II (SDHB) and complex IV (COXIV) were significantly higher in *Rev1*^−/−^ than in WT (Figure S3a), with a general trend towards increased expression of all respiratory complexes in liver from Rev1-deficient mice. However, some data on liver tissue from male mice were not statistically significant (Figs 2f,g, S3a,b). The increase in mtDNA content in the liver of *Rev1*^−/−^ mice is also not statistically significant (P = 0.089) (Figs 2h, S3c). In addition, expression of PGC1α was significantly lower in *Rev1*^−/−^ MEFs than in WT MEFs (Fig. 2d,e), but significantly higher in liver cells from female *Rev1*^−/−^ mice (Figures S3d and S5). Increased mitochondrial content was shown to be associated with a decrease in PGC1a and mitophagy^36^ We, therefore, tentatively conclude that the mitochondrial biogenesis is lower in Rev1-deficient MEFs, but higher in the liver of 5 months old *Rev1*^−/−^ mice (Figs 2f,i, S3d).

### Altered mitochondrial morphology and decreased mitophagy in *Rev1*^−/−^ cells

**Figure 3:**
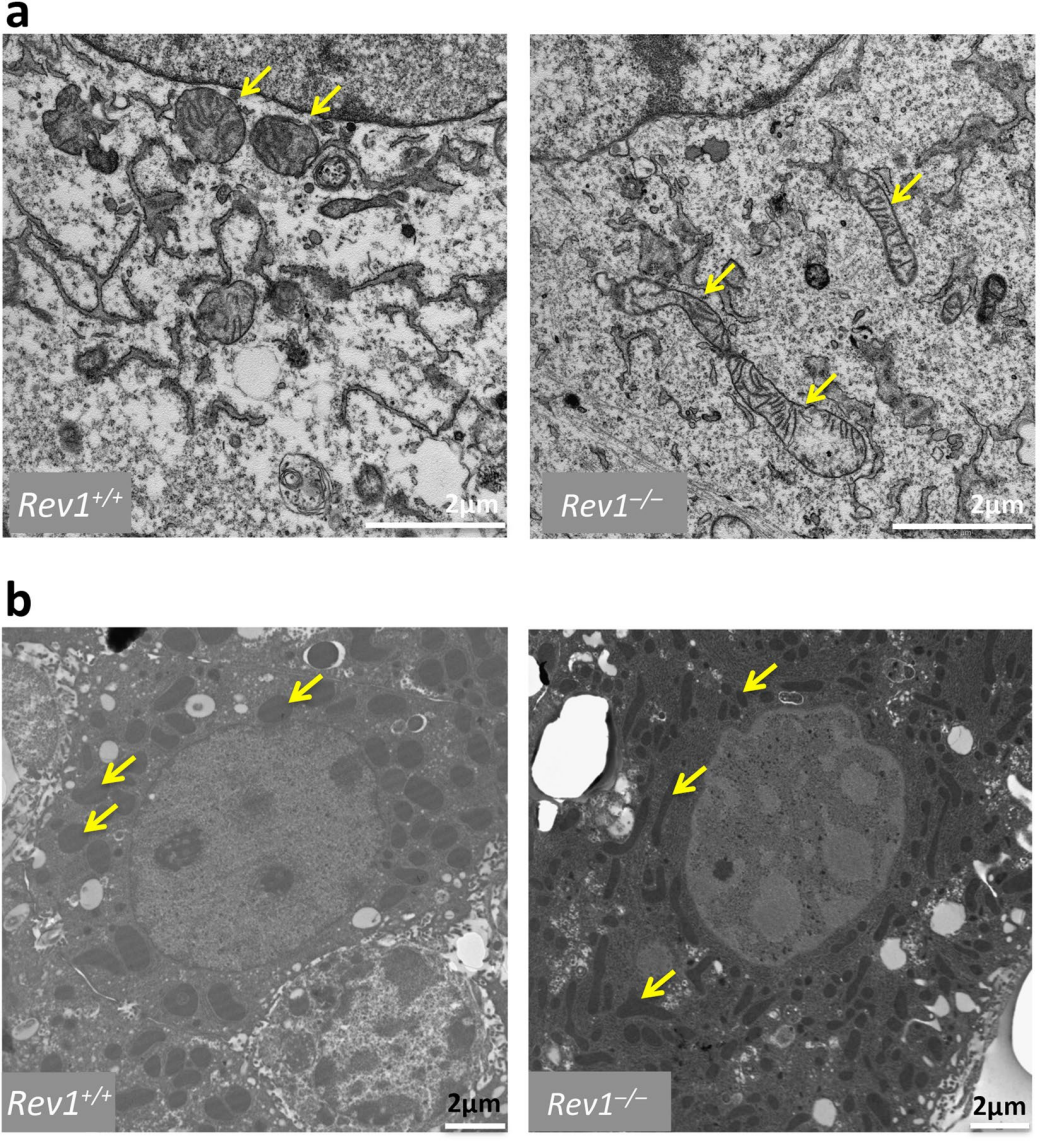
Analysis of mitochondrial morphology in MEF cells and liver hepatocytes. The TEM images of mitochondrial morphology in WT and *Rev1*^−/−^ MEF cells after 8 passages **(a)** and hepatocytes of 5 months old mice **(b)**. Size bars: 2 µM.

Change in mitochondrial morphology may reflect altered mitochondrial bioenergetics and/or increased energy demand^37^. Here, we compared mitochondrial morphology in WT and *Rev1*^−/−^ MEFs and hepatocytes using Transmission Electron Microscopy (TEM) (Fig. 3a,b). We observed fragmented and elongated mitochondria in *Rev1*^−/−^ hepatocytes and MEFs at a much greater frequency than in WT cells, where mitochondria were more homogenous in size and shape. One explanation might be that surveillance and maintenance of the mitochondrial pool is less efficient in Rev1-deficient cells; another possibility is that the rates of mitochondrial fission/fusion and/or mitophagy/autophagy are altered in *Rev1*^−/−^ cells^30,38–40^.

**Figure 4:**
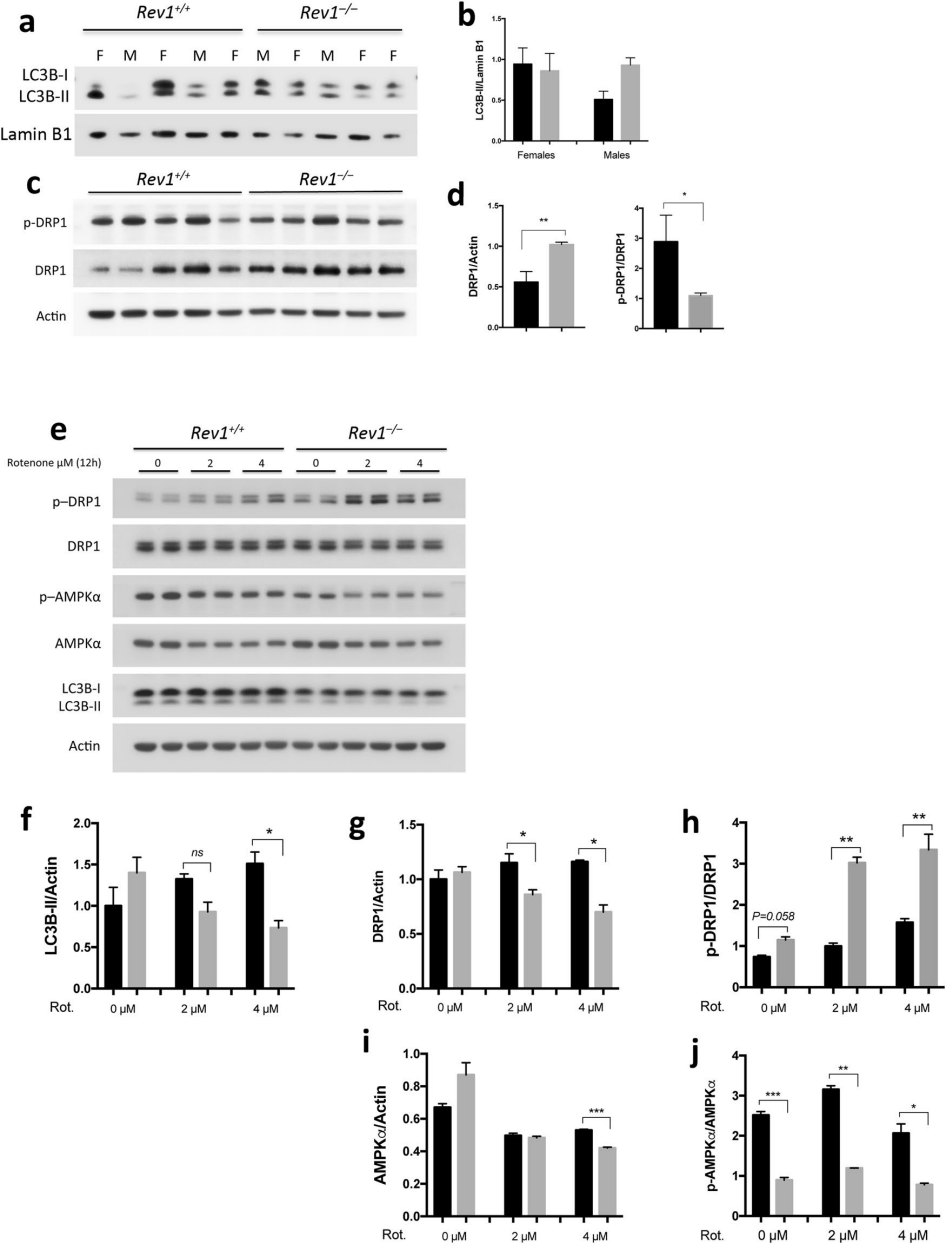
Analysis of autophagy and mitochondrial dynamics in MEFs and hepatocytes in WT and *Rev1*^−/−^ cells. (**a)** Analysis of autophagy by immunoblot and (**b)** quantification of LC3B-II/Actin ratio in the liver of WT and *Rev1*^−/−^ 5 months old mice (females, n = 3; males n = 2). (**c)** Immunoblot analysis of total and phosphorylated DRP1 (Ser616) in the liver of WT and *Rev1*^−/−^ 5 months old mice (male and females together, n = 5). (**d**) Quantification of total and phosphorylated DRP1 (Ser616) from panel (**c**). (**e**) Immunoblot with WT and *Rev1*^−/−^ MEF cells, treated with rotenone (0 μM, 2 µM and 4 µM) for 12 hrs (n = 3; df = 2). (**f)** The LC3B-II/Actin ratio in untreated and treated MEF cells after 12 hrs (0 µM: t = 1, 2 µM: t = 2.2, 4 µM: t = 14). (**g**,**h)** Analysis of total DRP1 (0 µM: t = 0.56; 2 µM: t = 5.1; 4 µM: t = 7.36) and phosphorylated DRP1 (Ser616) (0 µM: t = 3.96; 2 µM: t = 21.93; 4 µM: t = 14.14) in untreated and treated MEFs. (**i**,**j)** Analysis of total AMPKα (0 µM: t = 2.09; 2 µM: t = 0.56; 4 µM: 81.52) and phospho- AMPKα (Thr172) (0 µM: t = 64.37; 2 µM: t = 21.2; 4 µM: t = 4.67) in untreated and treated MEFs. n = sample number; t = t value; df = degree of freedom; *p < 0.05. **p < 0.01. ***p < 0.001. ****p < 0.0001. Data presented are mean ± M.S.E. Dark bars: WT and gray bars: *Rev1*^−/−^.

Autophagy is an intracellular process by which dysfunctional cellular components are degraded via the action of lysosomes. The rate of autophagy increases in response to starvation, growth factor deprivation, endoplasmic reticulum (ER) stress, infection, and other forms of stress^41^. Microtubule-associated protein 1 A/1B-light chain 3 (LC3)-related proteins are markers of autophagy, because of their critical roles in this pathway^41^. Cells initially synthesize the unprocessed polypeptide LC3-I, which is converted to LC3-II during the final stage of autophagy. Therefore, the rate at which LC3-I is converted to LC3-II reflects the rate of autophagy^42^. Here, we observed that the deduced rate of autophagy was similar in liver cells from WT and *Rev1*^−/−^ female mice (Fig. 4a,b), but appeared to be higher in liver cells from *Rev1*^−/−^ male mice than in control cells (Fig. 4a,b). Autophagy and mitophagy was also measured in MEFs after exposure to rotenone (2 or 4 μ μM) (Fig. 4e,f). Rotenone is a mitochondrial toxin, which inhibits complex I activity and induces mitochondrial specific autophagy (mitophagy)^36^. There were no significant differences in autophagy flux between untreated WT and *Rev1*^−/−^ MEFs. Untreated *Rev1*^−/−^ cells showed a slight increase in autophagy (Fig. 4f). In response to treatment with rotenone, the expression and conversion of LC3B-I to LC3B-II was decreased in *Rev1*^−/−^ cells, while it was increased in WT cells (Fig. 4e,f). To confirm this decrease in mitophagy in *Rev1*^−/−^ MEF cells, we assessed the intensity of mitophagy specific dye using confocal microscopy in live cells after treatment with rotenone (see methods for details) and observed that the intensity of mitophagy marker is significantly higher (*P < 0.0001*) in WT cells compared to *Rev1*^−/−^ MEFs (Figure S7). This suggests that stress-induced mitophagy may be impaired in Rev1-deficient cells.

The rate of mitochondrial fission was also examined in WT and Rev1-deficient cells^43^. Fission is a process through which mitochondria divide and give raise to smaller mitochondria. DRP1 is a GTPase member of the dynamin family, that oligomerizes on the surface of the outer mitochondrial membrane and promotes mitochondrial fission^44,45^. DRP1 is regulated via phosphorylation at Ser616 by cyclin dependent kinase (CDK1)/Cyclin B, which stimulates DRP1 activity and promotes efficient distribution of mitochondria to daughter cells during mitosis^46^. We measured total and Ser616-phosphorylated DRP1 (pDRP1 Ser616) (Fig. 4c–e) and observed a higher level of total DRP1 but a lower level of p-DRP1 Ser616 in the liver cells from *Rev1*^−/−^ mice (Fig. 4c,d). This result is consistent with suppressed DRP1 activity, less frequent mitochondrial fission and the presence of elongated mitochondria in Rev1-deficient cells. In contrast, DRP1 expression was similar in WT and *Rev1*^−/−^ MEFs. However, in response to treatment with rotenone, the total DRP1 protein is decreased in *Rev1*^−/−^ MEFs while it increased in WT (Fig. 4e,g). p-DRP1 Ser616 was higher in untreated *Rev1*^−/−^ than in untreated WT MEFs. The level of pDRP1 Ser616 increased significantly in rotenone treated WT and *Rev1*^−/−^ MEFs and these changes were greater in treated *Rev1*^−/−^ MEFs than in WT. We tentatively conclude that among the multiple factors that regulate mitochondrial dynamics and autophagy in MEFs, several are influenced by *Rev1* genotype^47,48^.

### Phosphorylation of AMPKα is decreased in Rev1^−/−^ cells

AMPK positively regulates autophagy through its ability to inhibit mechanistic target of rapamycin (mTOR), a negative regulator of autophagy. However, AMPK also phosphorylates ULK1 and Raptor, which promotes autophagy^47,49–51^. Active DRP1 binds to the mitochondrial fission receptor (MFF) and phosphorylation of MFF by AMPK is required for the oligomerization of DRP1 on the mitochondrial outer membrane^51^. In addition, AMPK is sensitive to the AMP:ATP ratio, and is considered to be a “metabolic sensor.” When the AMP:ATP ratio increases, the AMPK catabolic subunit, AMPKα is phosphorylated by LKB1 on Thr172, which stimulates its kinase activity. Activated AMPK phosphorylates downstream targets to decrease ATP consumption and promote mitochondrial biogenesis and ATP production^47^.

Here, we observed that while expression of AMPKα was higher the abundance of pAMPKα Thr172 was significantly lower in *Rev1*^−/−^ cells (Fig. 4e,i,j). 5-Aminoimidazole-4-carboxamide ribonucleotide (AICAR) is an analog of adenosine monophosphate (AMP) that stimulates AMPK activity. Therefore, WT and Rev1-deficient cells were treated with AICAR, and the effect on mitochondrial bioenergetics was investigated (Figure S4). The results show no significant change in basal- or ATP-linked respiration in *Rev1*^−/−^ or WT MEFs treated with AICAR relative to untreated controls. However, reserve capacity increased significantly in WT MEFs but decreased in *Rev1*^−/−^ MEFs relative to untreated cells. These data are consistent with the idea that Rev1-deficient cells display mitochondrial dysfunction and a defect in autophagy, which could reflect de-regulation of upstream signals including LKB1 and Sirtuin-1 (SIRT1).

### Rev1-deficiency affects mitochondrial function through PARP-NAD^+^-SIRT1-PGC1α axis

**Figure 5:**
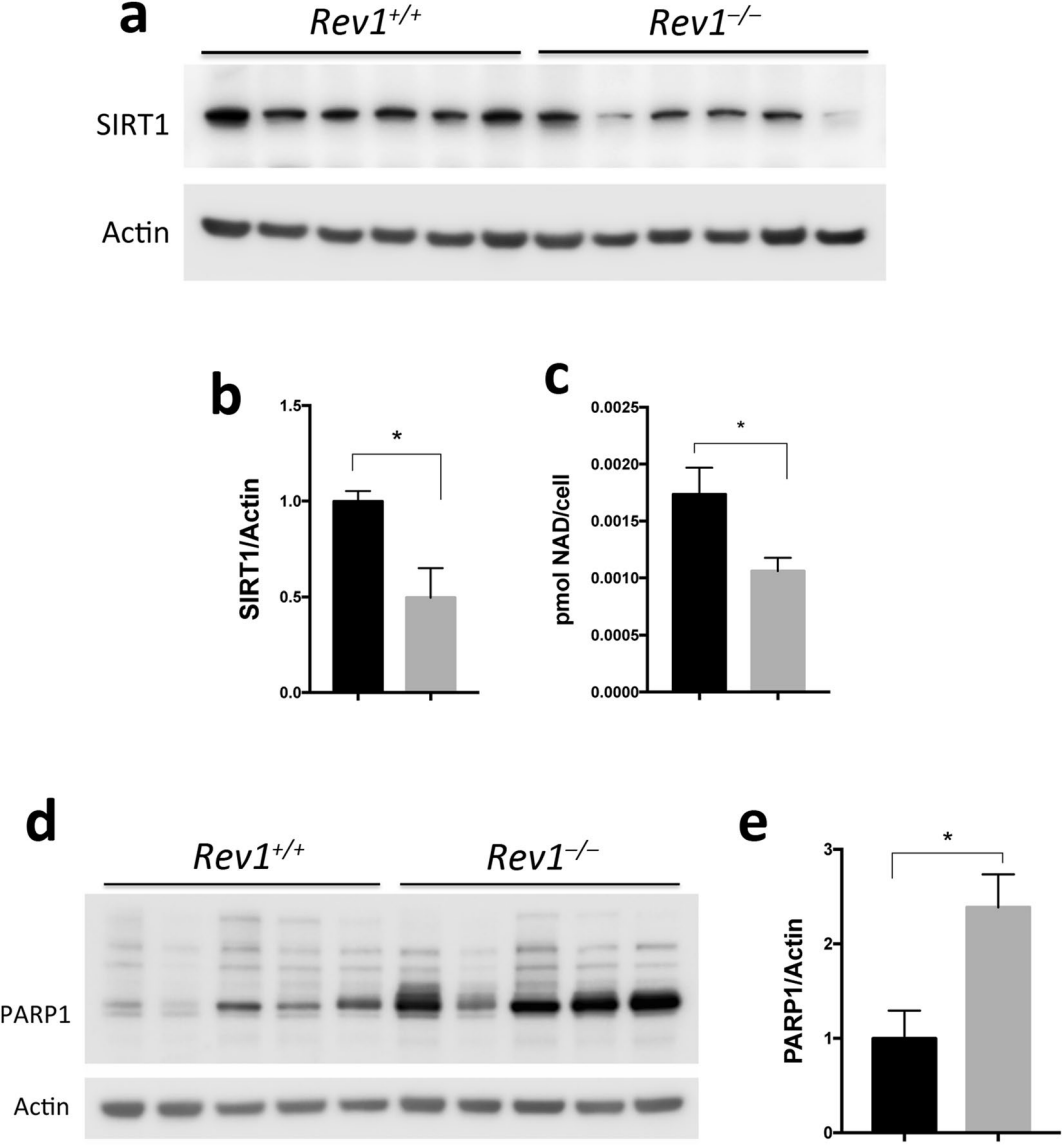
Cellular SIRT1 protein and NAD+ levels are decreased while the PARP1 protein level is increased in *Rev1*^−/−^ MEFs. (**a)** Immunoblot analysis of SIRT1 in MEFs in 6 biological replicates (n = 6; t = 3.457; df = 5). (**b)** Quantification of protein levels shown in panel **(a)**. (**c)** Relative NAD^+^ (NAD^+^/NADH) level in WT and *Rev1*^−/−^ MEFs (n = 6, t = 3.161, df = 10). (**d)** Immunoblot analysis of PARP1 in WT and *Rev1*^−/−^ MEF cell extracts in several biological replicates (n = 5, t = 4.251, df = 4). (**e)** Quantification of protein levels shown in panel **(d**). n = sample number; t = t value; df = degree of freedom; *p < 0.05. **p < 0.01. ***p < 0.001. ****p < 0.0001. Data presented are mean ± M.S.E. Dark bars: WT and gray bars: *Rev1*^−/−^.

SIRT1 is a class III histone deacetylase (HDAC), which requires NAD^+^ for activity. Deacetylation of target proteins by SIRT1 can either enhance or decrease their activity or stability^52^. SIRT1 deacetylates a number of target proteins including LKB1 and PGC1α. Deacetylation of LKB1 by SIRT1 promotes its activity and the phosphorylation of AMPK α at Thr172^52–55^. We investigated expression of SIRT1 in WT and *Rev1*^−/−^ MEFs and observed decreased abundance of SIRT1 in *Rev1*^−/−^ MEFs (Fig. 5a,b) as well as a significantly lower level of NAD^+^ in *Rev1*^−/−^ MEFs than in WT MEFs (Fig. 5c). These data suggest that low abundance of SIRT1 and NAD+ leads to low AMPK activity in Rev1-deficient cells.

PARP1 is a component of DDR that detects and binds to abnormal and damaged DNA^9,10,56^. Upon binding, PARP1 synthesizes and attaches poly(ADP-ribose) polymers (PAR) on its protein substrates. PARP1 also autoPARylates itself, thus, PARP1 can be a major consumer of intracellular NAD^+^^10,56,57^ and it can impair mitochondrial function^58^. The PARP1 expression is significantly increased in *Rev1*^−/−^ MEFs (Fig. 5d,e). To test whether decrease in NAD+ level is responsible for mitochondrial dysfunction in *Rev1*^−/−^ cells, we treated the MEF cells with 3 mM Nicotinamide riboside (NR), a NAD+ precursor (Figure S8). NR treatment enhanced mitochondrial function and increased the reserve capacity to the level of untreated *Rev1*^*+/+*^ cells. This suggests that impaired mitochondrial function in *Rev1*^*+/+*^ cells is caused by NAD^+^ depletion and NR supplement can suppress this defect.

Here, we propose that defects in *Rev1* could increase the load of persistent DNA lesions and thus lead to constitutive activation of PARP1. It has previously been proposed that such a pathway reflects “nuclear to mitochondrial DNA damage signaling”^7^. Our results are consistent with activation of such a pathway in Rev1-deficient cells, because the abundance of PARP1 is significantly higher in *Rev1*^−/−^ MEFs than in WT MEFs (Fig. 5d,e).

## Discussion

**Figure 6:**
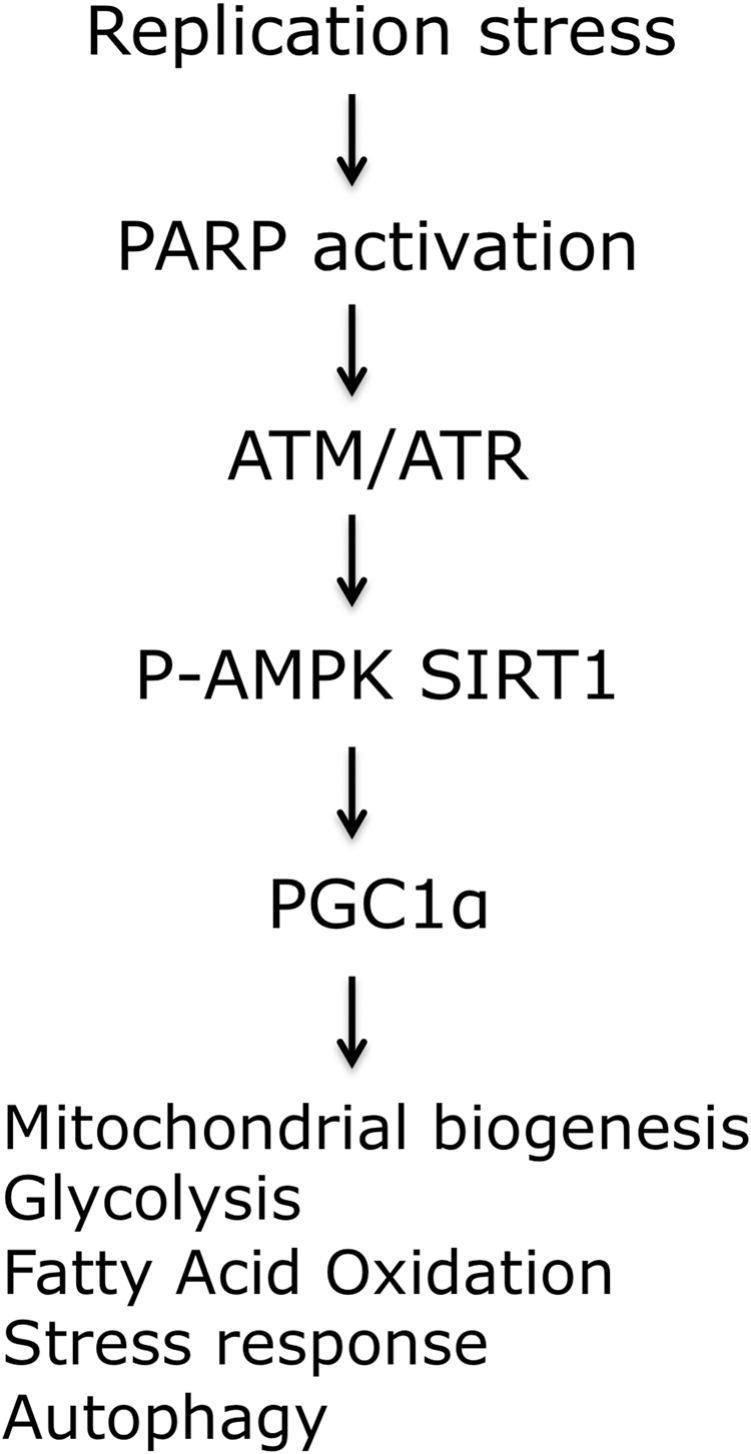
The “PARP-NAD^+^-SIRT1-PGC1α axis”. Replication stress can cause mitochondrial dysfunction by downstream changes in various cellular pathways. A number of factors contribute to this signaling, such as poly(ADP-ribose) polymerase 1 (PARP1), ataxia telangiectasia mutated (ATM), ataxia telangiectasia and Rad3-related protein (ATR), and the transcription factor p53. The NAD-dependent protein deacetylase sirtuin 1 (SIRT1) and the transcription regulator AMP-activated protein kinase (AMPK) then propagate the signal through post-translational modifications (deacetylation or phosphorylation, respectively) of peroxisome proliferator-activated receptor-γ co-activator 1α (PGC1α) and other proteins. The figure is adapted from Fang *et al*. 2016^7^.

This study examines whether and how murine Rev1 might play a direct role in or modulate mitochondrial function(s) and cellular bioenergetics. Our data are consistent with the idea that Rev1-deficiency leads to replicative stress, which may in turn lead to constitutive activation of DDR and PARP1, decreased abundance of NAD^+^, and low SIRT1 and AMPK activities. Similar observations were recently made in other DNA repair deficient cells, and this set of related observations was referred to in that context as the “PARP-NAD^+^-SIRT1-PGC1α axis”^7^ (Fig. 6).

Rev1-deficient cells are unable to bypass the damaged bases and replication stress is higher in these cells^59^. *Rev1*^−/−^ cells accumulate single stranded gaps opposite the DNA lesions, which induce DNA damage signaling and irreversible G2 arrest^13^. In response to DNA damage and DDR activation, cells increase mitochondrial respiration and OXPHOS activity to increase ATP^60^.

The altered mitochondrial morphology, decreased mitophagy, increased basal and ATP linked respiration as well as increased MMP and mitochondrial ROS in *Rev1*^−/−^ suggests that ATP production may be higher in these cells than in WT controls,^30,31,37,61^. However, we observed that total ATP was nearly 2-fold lower in *Rev1*^−/−^ than in WT control cells. This indicates that ATP consumption exceeds ATP production in *Rev1*^−/−^ MEFs as these cells are constantly under replication stress^59^. In addition, we observed that the PARP1 expression is increased and the NAD^+^ level is decreased in Rev1-deficient cells, which can impair mitochondrial function, mitophagy and ATP synthesis^7,11,58^. Interestingly, we could suppress the decreased mitochondrial reserve capacity in *Rev1*^−/−^ MEFs by addition of the NAD+ precursor NR (Figure S8). This supports the axis, Fig. 6, where the decreased levels of NAD lead to mitochondrial dysfunction, and this can at least partially be restored by NAD supplementation.

Despite a significant decrease in abundance of ATP, AMPK activity was lower in *Rev1*^−/−^ cells. This is an important observation, because AMPK is required to inhibit Akt–mTOR^62,63^, and to activate mitophagy/autophagy in response to replicative or other forms of chronic stress. In addition, increased Akt activity negatively regulates the SOD2 expression^64^.

Despite a significant increase in total DRP1 and pDRP1 ser616 in Rev1-deficient MEFs, these cells appear to accumulate elongated mitochondria with aberrant morphological features. This could reflect the low level of activated AMPK in Rev-1 deficient cells, because activated AMPK stimulates MFF, promotes oligomerization of DRP1 on the surface of mitochondria, and stimulates mitophagy^51^. Decreased AMPKα phosphorylation can be explained by the decrease in SIRT1 protein level and perhaps its activity as NAD^+^ level was decreased in *Rev1*^−/−^ MEFs^53–55^. As treatment with AICAR did not rescue mitochondrial dysfunction in Rev1-deficient cells, we conclude that signals downstream of AMPK, such as SIRT1 and LKB1, may be suppressed or dysfunctional in *Rev1*^−/−^ MEFs.

In conclusion, the results presented here suggest that Rev1-deficient MEFs and liver tissue may experience persistent replication stress and DDR, which leads to metabolic stress caused by mitochondrial dysfunction, mediated by the so-called “PARP-NAD^+^-SIRT1-PGC1α axis”^7^ (Fig. 6). In addition, our results suggest that AMPK may also tie into the “PARP-NAD^+^-SIRT1-PGC1α axis”, and suppression of its activity could exacerbate mitochondrial dysfunction in these cells.

## Materials and Methods

### Animals and Cells

WT and *Rev1*^−/−^ mice and MEF cells were provided by Dr. Niels de Wind from the department of human genetics at Leiden University Medical Center (LUMC). All procedures involving animals were approved by the Danish National Ethics Committee according to the guidelines set forth in the European Council’s Convention for the Protection of Vertebrate Animals used for Experimental and Other Scientific Purposes. Mice were randomized and marked with special ear cuts for later identification. WT and *Rev1*^−/−^ mice and MEFs were genotyped as described before^65^. We excluded mouse 7 (due to being heterozygous) and 9 (for being sick and moribund) (Figure S5a). Sex determination by PCR in mice was performed as described^66^. Mice were sacrificed by cervical dislocation and the liver, kidneys, heart and brain were removed. MEF cells cultured in Dulbecco’s Modified Eagle Medium (DMEM) plus 10% (v/v) fetal calf serum (Invitrogen) and 100 U/mL penicillin-streptomycin in a humidified atmosphere of 5% CO2 at 37 °C. All the experiments are carried out latest two weeks after the initiation of the cell culture or as stated.

### Isolation of Mitochondria from the Mice Liver

Mitochondria from WT and *Rev1*^−/−^ mice aged 5 months were isolated by centrifugation methods as described^67^. The liver was extracted and sliced in MSHE+ BSA (4 °C), and all subsequent steps of the preparation were performed on ice. The material was rinsed several times to remove blood. The tissue was disrupted using a drill-driven Teflon glass homogenizer with 2–3 strokes. Homogenate was centrifuged at 800 × g for 10 min at 4 °C. Following centrifugation, fat/lipid was carefully removed, and the remaining supernatant was decanted through 2 layers of cheesecloth to a separate tube and centrifuged at 8000 × g for 10 min at 4 °C. After removal of the mitochondrial layer, the pellet was resuspended in MSHE+ BSA, and the centrifugation was repeated. The final pellet was resuspended in a minimal volume of MSHE+ BSA. Total protein (mg/ml) was determined using Bradford Assay reagent (Bio-Rad).

### Cellular bioenergetics and citrate synthase assay

All XF assays were performed using an XF24 Extracellular Flux Analyzer (Seahorse Bioscience):

### MEF cells

Oxygen consumption rate and cellular acidification rate in MEF cells measured according to the manufacturer procedure. Briefly, 5 × 10^4^ cells/well seeded on the seahorse plate 12 hrs before the experiment. For the AICAR treatment, cells were treated with AICAR (0.25 mM) for 96 hrs prior to the experiment. Total protein was extracted from the cells in each well and protein concentrations determined by Bradford. The basal respiration was normalized to the protein content of each well.

#### Isolation of mitochondria from liver

Respiration of isolated liver mitochondria was determined with a Seahorse XF24 Analyzer. The isolation procedure was performed on ice. Liver was excised and immersed in ice-cold mitochondrial isolation buffer (MSHE; in mM: sucrose 70; mannitol 210; HEPES 5 and EGTA 1, pH 7.0) + 0.5% (w/v) fatty acid-free BSA and homogenized in a dounce type teflon-on-glass homogenizer. The homogenate was centrifuged 5 min at 900×*g*. The supernatant was collected, while avoiding top layer of fat, and transferred to Eppendorf tubes and centrifuged for 10 min at 9000×*g*. Following centrifugation, fat was carefully removed and pellet was resuspended in MSHE buffer where after the suspension was centrifuged for 10 min at 10,000×*g*. The pellet was re-suspended in MSHE. Protein concentration was determined spectrophotometrically at 595 nm by addition of 5 μl diluted mitochondrial suspension and 250 μl Bio-Rad (Hercules, CA, USA) Protein Assay Dye reagent. BSA was used as standard protein.

#### Determination of parameters of oxidative phosphorylation of isolated liver mitochondria

The suspension of isolated mitochondria was diluted in supplemented MSHE (10 mM succinate +2 μM rotenone for coupling assay and 10 mM pyruvate +2 mM malate +4 μM FCCP for electron flow assay) and added to a final concentration of 5 μg to each well in a 24-well Seahorse plate. The plate was centrifuged at 2,000×*g* for 10 min at RT. Mitochondrial assay solution (MAS; in mM: sucrose 70; mannitol 220; KH_2_PO_4_ 10; MgCl_2_ 5; HEPES 2 and EGTA 1; pH 7.0) was added and the plate was transferred to the XF24 Seahorse at 37 °C. For monitoring mitochondrial respiration the measurement cycle consisted of 1 min mixing and one measurement cycle (each measurement cycle reflects an average of ten measurements) before addition of first inhibitor/substrate compound (40 mM ADP for coupling assay, 20 µM rotenone for electron flow assay), then another measurement cycle and injection of second compound (3 μM oligomycin for coupling assay, 0.1 M succinate for electron flow assay), and another two cycles after injection of third compound (4 µM FCCP for coupling assay, 40 μM antimycin A for electron flow assay), and fourth compound (40 μM antimycin A for coupling assay, 100 mM ascorbate +1 mM TMPD for electron flow assay) respectively. Each point represents an average of a minimum of 3 replicate wells and each condition was repeated a minimum of 5 times.

#### Citrate synthase activity assay on liver homogenate

The citrate synthase activity (CS) assay was determined as described^68^ with minor modifications. All steps were done on ice and centrifugation steps at 4 °C. Liver tissue was immersed in ice cold MSHE buffer after excision, and washed with MSHE buffer to remove blood. The liver tissue was snap frozen in liquid nitrogen and stored at −80 °C. 30–50 mg of tissue was used for CS assay. Visible fat was removed and liver tissue was minced with scissors before dilution in 5 ml MSHE +0.5% fatty acid free BSA buffer in a 1-ml douncer with a teflon-on-glass homogenizer. The homogenate was centrifuged at 10 min for 600×*g*, and the supernatant was transferred to a new tube and centrifuged at 10 min for 7000×*g*. The supernatant was discarded and pellet resuspended in remaining buffer. In a 1-ml quartz cuvette the following things were mixed: 300 μl distilled water, 500 μl Tris (200 mM, pH 8.0) with 0.2% Triton X-100 (v/v), 100 μl DTNB (5,5′-Dithiobis(2-nitrobenzoic acid, 1mM), 30 μl of Acetyl-coenzyme A lithium salt (10 mM) and 60 μg liver tissue. The baseline activity was read at absorbance 412 nm for 2 min, hereafter the reaction was started by 50 μl of oxaloacetic acid 10 mM, and the increase in absorbance was monitored for 3 min. Activity was measured as ((ΔAbsorbance/min) × 1000)/(13.6 × sample(ml) × protein concentration (mg/ml). All the reagents were from Sigma-Aldrich.

### Isolation of genomic DNA and mtDNA quantification

Genomic DNA (gDNA), RNA and protein were extracted from snap frozen tissues in liquid nitrogen, using Norgen Biotik RNA/DNA Purification Kit (Cat. 48700) according to the manufacturer’s protocol. Quantitative PCR (two independent reactions) was used for measurement of mtDNA^69^ using SYBR Green PCR Master mix (Applied Biosystems). The mtDNA copy number normalized to nuclear DNA was measured as the ratio of DNA encoding the mitochondrial gene for murine tRNA leucine 1 (Trnl1) to DNA encoding the nuclear gene encoding Beta-2 microglobulin (B2m). The following primers were used: B2m forward, 5′-GTCAGATATGTCCTTCAGCAAG-3′, and reverse, 5′-CTTcAACTCTGCAGGCGTATG-3′; and Trnl1 forward, 5′-AAGGTTATTAGGGTGGCAG-3′, and reverse, 5′-GGACGAGGAGTGTTAGGATA-3′. For PCR reaction mixture 5 ng of genomic DNA was mixed with 2 μl of each primers (5 μM), 10 μl SYBR green and 0.4 μl ROX and nuclease free water to the final volume of 20 μl. All reactions were run in triplicates. The relative mtDNA levels were calculated using formula 2 × 2^ΔCT^.

### Immunoblot analysis

The frozen liver tissues were ground in liquid nitrogen prior to incubation with RIPA (Sigma) supplemented with supplemented with cOmplete™, EDTA-free Protease Inhibitor Cocktail (Sigma) and PhosSTOP phosphatase inhibitor Cocktail (Sigma). MEFs cells and grinded tissue were re-suspended in RIPA (Sigma) supplemented with supplemented with cOmplete™, EDTA-free Protease Inhibitor Cocktail (Sigma) and PhosSTOP phosphatase inhibitor Cocktail (Sigma). The cell suspension was left on ice for 30 min followed by sonication and then left on ice for another 15 min. Subsequently, the cell suspension was centrifuged at 20K G-force for 20 min and the supernatant was mixed with NuPAGE® LDS Sample Buffer (4X) (Thermo Fisher Scientific). For gel electrophoresis, the supernatants was denatured at 70 °C (50 °C for mitochondrial complexes analyses) for 10 min and 20 to 30 μg protein was loaded on NuPAGE™ Novex™ 4–12% Bis-Tris Protein Gels, 1.0 mm (Thermo Fisher Scientific) and run according to the manufacturer’s instructions.

Antibodies used in this study: pan-Actin (Thermo Fisher Scientific MA5-11869) (1:20000); AMPKα1 (Santa Cruz bio. Sc-19128) (1:500); Phospho-AMPKα (Thr172)(Cell signaling #2531) (1:1000); COX4 (Santa Cruz bio. Sc-69360) (1:2000); DRP1 (Cell signaling #8570) (1:1000); Phospho-DRP1 (Ser616) (Cell signaling #3455) (1:1000); Lamin B1 (Abcam ab133741) (1:10000); LC3B/MAP1LC3B (Novus Biologicals, NB100-2220) (1:1000); Total OXPHOS Rodent WB Antibody Cocktail (Abcam, ab110413) (1:1000); PARP (Cell signaling, #9542) (1:1000); PGC1α (Novus Biologicals, NBP1-04676) (1:3000); SIRT1 (Santa Cruz bio, sc-15404) (1:500); SOD2 (Cell signaling, #13194) (1:1000).

### Measurements mitochondrial membrane potential and ROS

Mitochondrial membrane potential and ROS were measured as described elsewhere using MitoSOX Red and TMRM (Thermo Fisher Scientific) respectively^70^. Briefly, 0.7 × 10^5^ cells/well were seeded in six well plates less than 48 hrs before initiation of the experiment. Cells were washed with Dulbecco’s Phosphate-Buffered Saline (DPBS) and treated with 5 µM MitoSOX Red for 15 min and 40 µM TMRM for 20 min prior to FACS analysis. MitoSOX Red-positive cells were counted using a FACScanTM flow cytometer (Becton Dickinson). For live cell fluorescent imaging of MMP, approximately 2 × 10^5^ cells/6cm culture dish seeded 24h before the experiment. Treated cell were incubated with 2 µM rotenone for 5h in complete medium. After treatment cells were washed with PBS and incubated with 40 nM TMRM in serum free medium for 30 minutes. After staining cells were washed and incubated with serum free media for live cell imaging using confocal microscopy (Zeiss Confocal microscope LSM 780).

### ATP and NAD^+^/NADH Quantitation

ATP concentration was detected using an ATPlite Luminescence Assay System (PerkinElmer) according to the manufacturer’s instruction. Briefly, cells were plated in a 96-well plate (10^5^ cells in 100 ml/well) for 24 hrs before the ATP consumption assay. After this time, 50 µl lysis buffer was added followed by addition of substrate. Luminescence was measured on the FLUOstar OPTIMA Microplate reader (BMG LABTECH). Measurement of NAD^+^ is performed using SIGMA NAD/NADH Quantitation kit (MAK037) according to the manufacturer protocol. Briefly, 2 × 10^4^ cells harvested for total NAD extraction and quantification. The NAD Cycling Enzyme Mix in the kit recognizes NADH and NAD, but not NADP or NADPH, in an enzyme cycling reaction. Concentration of NAD total (NADt) (NAD^+^ + NADH) is determined by measuring the absorbance at (OD 450 nm). The NAD^+^ concentration is calculated by subtracting the NADH values from NADt. In Some *Rev1*^−/−^ samples, the NADH concentration was below the detection range. We perceived the NADH concentration in these samples as zero (0).

### Colony forming assay

For the cell survival assay, 700 cells were seeded in 100 mm culture dishes. After 24 hrs, different concentrations of menadione (0, 5, 10, 15, and 20 μM) were added to the cell culture. The cultures were maintained until colonies were visible. The colonies were then fixed and stained with crystal violate in 20% ethanol, and colonies with more than 50 cells, were counted as a colony.

### Detection of Autophagy and Mitophagy

Analysis of autophagy/mitophagy was carried out by detecting the LC3-II/actin ratio^71^. Briefly, 7 × 10^5^ cells/dish were seeded on 10 cm cell culture dishes. The following day, mitophagy was induced by treatment with 2 and 4 μM rotenone for 12 hrs. After this time, cells were harvested and prepared for immunoblotting. We used a commercially mitophagy kit (Dojindo Laboratories) to assess the rate of mitophagy as described by provider. About 2 × 10^5^ cells were seeded on cover slips in 6 cm culture dishes 24 h before the live imaging. The next day, cells were washed twice with PBS and serum free media. The mitophagy dye diluted to the recommended working concentration (100 nM/l) in serum free media and cells incubated for 30 min. The media and dye was removed and cells were washed twice with PBS and serum free media. Next, Cells were incubated with complete media containing 2 µM rotenone for 5h. After 5h, the intensity of mitophagy dye was asses using confocal microscopy (Zeiss Confocal microscope LSM 780). Pictures were analyzed using image J software.

### Transfection, fluorescence microscopy

Transfection of the YFP-Rev1 fusion protein into *Rev1*^−/−^ MEF cells was performed using Lipofectamine^®^ 3000 reagent (Life technologies) according to the manufacturer procedure. Briefly, 3 × 10^5^ cells/well were seeded in 6 well plates 18 hrs before the transfection. Cells were transfected with 2.5 μg of plasmid DNA per well in complete medium. Transfected cells were visualized with an inverted fluorescence microscope (Nikon). MitoTracker staining (Molecular Probes) was performed in live MEFs and analyzed by fluorescent microscopy after fixation with 4% formaldehyde.

### Electron microscopy

#### Mice Livers

For transmission electron microscopy studies small blocks (typically 0.5 mm × 0.5 mm × 1 mm) from the liver from WT and *Rev1*^−/−^ mice, perfusion-fixed with 4% paraformaldehyde and 0.1 glutaraldehyde in phosphate buffer were cryoprotected in glycerol and freeze substituted with Lowicryl HM20 as described^72^. Ultrathin sections were cut (90 nm) using a diamond knife and dried at room temperature followed by counterstaining with 1% uranyl acetate and 0.3% lead citrate. Electron micrographs were taken with a FEI Tecnai 12 transmission electron microscope at primary magnifications of ×6000.

#### MEF Cells

Cells were seeded on the Thermanox™ coverslips (Thermoscientific) in 12 well plates at the concentration of 5 × 10^4^ cells/well. When the cells became confluent, they were washed with PBS, and fixed in 2% glutaraldehyde/0.1 M sodium cacodylate buffer (pH 7.4) at room temperature for 1 hr. Cells were washed twice in PBS and postfixed in 2% OsO4/PBS at room temperature for 1 hr, dehydrated in ethanol and embedded in Epon (Serva, Heidelberg, Germany). Thin sections of 50 nm were contrasted with uranylacetate and lead citrate, and examined using a transmission electron microscope (Tecnai G2 20 TWIN).

### Statistical analysis

Prism 7.0 (GraphPad Software) was used to plot data, make graphs, and statistical analysis. Data are plotted as mean ± SEM. Two-tailed paired and unpaired Student’s t-test was performed for MEFs and liver cells.

## Electronic supplementary material

Supplementary Dataset 1

## References

[1] Burcham, P. C. Internal hazards: baseline DNA damage by endogenous products of normal metabolism (1999). 10.1016/s1383-5742(99)00008-3 10415429

[2] Schiewer MJ, Knudsen KE (2016). Linking DNA Damage and Hormone Signaling Pathways in Cancer. Trends Endocrinol. Metab..

[3] Sancar A, Lindsey-Boltz La, Unsal-Kaçmaz K, Linn S (2004). Molecular mechanisms of mammalian DNA repair and the DNA damage checkpoints. Annu. Rev. Biochem..

[4] Zeman MK, Cimprich Ka (2014). Causes and consequences of replication stress. Nat. Cell Biol..

[5] Bakkenist CJ, Kastan MB (2004). Initiating Cellular Stress Responses. Cell.

[6] Ward I, Chen J (2004). Early events in the DNA damage response. Curr. Top. Dev. Biol..

[7] Fang EF (2016). Nuclear DNA damage signalling to mitochondria in ageing. Nat. Rev. Mol. Cell Biol..

[8] Murphy MP (2009). How mitochondria produce reactive oxygen species. Biochem. J..

[9] Caldecott K (2007). Mammalian single-strand break repair: Mechanisms and links with chromatin. DNA Repair (Amst)..

[10] De Vos M, Schreiber V, Dantzer F (2012). The diverse roles and clinical relevance of PARPs in DNA damage repair: current state of the art. Biochem. Pharmacol..

[11] Cantó C, Menzies KJ, Auwerx J (2015). NAD(+) Metabolism and the Control of Energy Homeostasis: A Balancing Act between Mitochondria and the Nucleus. Cell Metab..

[12] Waters LS, Walker GC (2006). The critical mutagenic translesion DNA polymerase Rev1 is highly expressed during G(2)/M phase rather than S phase. Proc. Natl. Acad. Sci. USA.

[13] Jansen JG (2009). Separate Domains of Rev1 Mediate Two Modes of DNA Damage Bypass in Mammalian Cells. Mol. Cell. Biol..

[14] Friedberg EC, Lehmann AR, Fuchs RPP (2005). Trading Places: How Do DNA Polymerases Switch during Translesion DNA Synthesis?. Mol. Cell.

[15] Lehmann AR (2005). Replication of damaged DNA by translesion synthesis in human cells. FEBS Lett..

[16] Yoon JH (2015). Rev1 promotes replication through UV lesions in conjunction with DNA polymerases η, ι, and κ but not DNA polymerase ζ. Genes Dev..

[17] Dumstorf CA, Mukhopadhyay S, Krishnan E, Haribabu B, McGregor WG (2009). REV1 Is Implicated in the Development of Carcinogen-Induced Lung Cancer. Mol. Cancer Res..

[18] Xu H-L (2013). Effects of polymorphisms in translesion DNA synthesis genes on lung cancer risk and prognosis in Chinese men. Cancer Epidemiol..

[19] Goričar K (2015). Translesion polymerase genes polymorphisms and haplotypes influence survival of osteosarcoma patients. OMICS.

[20] Tomas-Roca L (2015). De novo mutations in PLXND1 and REV3L cause Möbius syndrome. Nat. Commun..

[21] Zhang, H., Chatterjee, A. & Singh, K. K. Saccharomyces cerevisiae Polymerase z Functions in Mitochondria. **2688**, 2683–2688 (2006). 10.1534/genetics.105.051029 PMC1456388 16452144

[22] Tsaalbi-Shtylik A (2015). Excision of translesion synthesis errors orchestrates responses to helix-distorting DNA lesions. J. Cell Biol..

[23] Jansen JG, Tsaalbi-Shtylik A, de Wind N (2015). Roles of mutagenic translesion synthesis in mammalian genome stability, health and disease. DNA Repair (Amst)..

[24] Kalifa L, Sia EA (2007). Analysis of Rev1p and Pol zeta in mitochondrial mutagenesis suggests an alternative pathway of damage tolerance. DNA Repair (Amst)..

[25] Baruffini E (2012). Overexpression of DNA Polymerase Zeta Reduces the Mitochondrial Mutability Caused by Pathological Mutations in DNA Polymerase Gamma in Yeast. PLoS One.

[26] Rasmussen AK, Chatterjee A, Rasmussen LJ, Singh KK (2003). Mitochondria-mediated nuclear mutator phenotype in Saccharomyces cerevisiae. Nucleic Acids Res..

[27] Andersen PL, Xu F, Ziola B, McGregor WG, Xiao W (2011). Sequential assembly of translesion DNA polymerases at UV-induced DNA damage sites. Mol. Biol. Cell.

[28] Kim H, Yang K, Dejsuphong D, D’Andrea AD (2012). Regulation of Rev1 by the Fanconi anemia core complex. Nat. Struct. Mol. Biol..

[29] Brand MD, Nicholls DG (2011). Assessing mitochondrial dysfunction in cells. Biochem. J..

[30] Gomes LC, Di Benedetto G, Scorrano L (2011). During autophagy mitochondria elongate, are spared from degradation and sustain cell viability. Nat. Cell Biol..

[31] Liesa M, Shirihai OS (2013). Mitochondrial dynamics in the regulation of nutrient utilization and energy expenditure. Cell Metab..

[32] Weisiger RA, Fridovich I (1973). Mitochondrial superoxide disimutase. Site of synthesis and intramitochondrial localization. J. Biol. Chem..

[33] Valko M (2007). Free radicals and antioxidants in normal physiological functions and human disease. Int. J. Biochem. Cell Biol..

[34] Fukai T, Ushio-Fukai M (2011). Superoxide dismutases: role in redox signaling, vascular function, and diseases. Antioxid Redox Signal.

[35] Monks TJ, Hanzlik RP, Cohen GM, Ross D, Graham DG (1992). Quinone chemistry and toxicity. Toxicol. Appl. Pharmacol..

[36] Fang EF (2014). Defective Mitophagy in XPA via PARP-1 Hyperactivation and NAD+/SIRT1 Reduction. Cell.

[37] Westermann B (2012). Bioenergetic role of mitochondrial fusion and fission. Biochim. Biophys. Acta - Bioenerg..

[38] Twig G (2008). Fission and selective fusion govern mitochondrial segregation and elimination by autophagy. EMBO J..

[39] Okamoto K, Kondo-Okamoto N (2012). Mitochondria and autophagy: critical interplay between the two homeostats. Biochim. Biophys. Acta.

[40] Gomes LC, Scorrano L (2013). Mitochondrial morphology in mitophagy and macroautophagy. Biochim. Biophys. Acta.

[41] He C, Klionsky DJ (2009). Regulation mechanisms and signaling pathways of autophagy. Annu. Rev. Genet..

[42] Klionsky, D. J. *et al*. Guidelines for the use and interpretation of assays for monitoring autophagy (3rd edition) **8627** (2016). 10.1080/15548627.2015.1100356 PMC4835977 26799652

[43] Youle RJ, van der Bliek AM (2012). Mitochondrial fission, fusion, and stress. Science.

[44] Yoon Y, Pitts KR, McNiven MA (2001). Mammalian dynamin-like protein DLP1 tubulates membranes. Mol. Biol. Cell.

[45] Chan DC (2012). Fusion and fission: interlinked processes critical for mitochondrial health. Annu. Rev. Genet..

[46] Taguchi N, Ishihara N, Jofuku A, Oka T, Mihara K (2007). Mitotic phosphorylation of dynamin-related GTPase Drp1 participates in mitochondrial fission. J. Biol. Chem..

[47] Mihaylova MM, Shaw RJ (2011). The AMPK signalling pathway coordinates cell growth, autophagy and metabolism. Nat. Cell Biol..

[48] Zhang C-S, Lin S-C (2016). AMPK Promotes Autophagy by Facilitating Mitochondrial Fission. Cell Metab..

[49] Kim J, Kundu M, Viollet B, Guan K-L (2011). AMPK and mTOR regulate autophagy through direct phosphorylation of Ulk1. Nat. Cell Biol..

[50] Alers S, Löffler AS, Wesselborg S, Stork B (2012). Role of AMPK-mTOR-Ulk1/2 in the regulation of autophagy: cross talk, shortcuts, and feedbacks. Mol. Cell. Biol..

[51] Toyama EQ (2016). AMP-activated protein kinase mediates mitochondrial fission in response to energy stress. Science (80-.)..

[52] Houtkooper RH, Pirinen E, Auwerx J (2012). Sirtuins as regulators of metabolism and healthspan. Nat. Rev. Mol. Cell Biol..

[53] Hou X (2008). SIRT1 regulates hepatocyte lipid metabolism through activating AMP-activated protein kinase. J. Biol. Chem..

[54] Lan F, Cacicedo JM, Ruderman N, Ido Y (2008). SIRT1 modulation of the acetylation status, cytosolic localization, and activity of LKB1. Possible role in AMP-activated protein kinase activation. J. Biol. Chem..

[55] Price NL (2012). SIRT1 is required for AMPK activation and the beneficial effects of resveratrol on mitochondrial function. Cell Metab..

[56] Hakmé A, Wong H-K, Dantzer F, Schreiber V (2008). The expanding field of poly(ADP-ribosyl)ation reactions. ‘Protein Modifications: Beyond the Usual Suspects’ Review Series. EMBO Rep..

[57] Sims JL, Berger SJ, Berger NA (1981). Effects of nicotinamide on NAD and poly (ADP-ribose) metabolism in DNA-damaged human lymphocytes. J. Supramol. Struct. Cell. Biochem..

[58] Bai P, Nagy L, Fodor T, Liaudet L, Pacher P (2015). Poly(ADP-ribose) polymerases as modulators of mitochondrial activity. Trends Endocrinol. Metab..

[59] Martín-Pardillos, A. *et al*. Genomic and functional integrity of the hematopoietic system requires tolerance of oxidative DNA lesions. *Blood* In press (2017). 10.1182/blood-2017-01-764274 PMC5620415 28827409

[60] Qin L (2015). CDK1 Enhances Mitochondrial Bioenergetics for Radiation-Induced DNA Repair. Cell Rep..

[61] Nicholls DG (2002). Mitochondrial function and dysfunction in the cell: its relevance to aging and aging-related disease. Int. J. Biochem. Cell Biol..

[62] Zoncu R, Efeyan A, Sabatini DM (2011). mTOR: from growth signal integration to cancer, diabetes and ageing. Nat. Rev. Mol. Cell Biol..

[63] Inoki K, Kim J, Guan K-L (2012). AMPK and mTOR in cellular energy homeostasis and drug targets. Annu. Rev. Pharmacol. Toxicol..

[64] Eijkelenboom A, Burgering BMT (2013). FOXOs: signalling integrators for homeostasis maintenance. Nat. Rev. Mol. Cell Biol..

[65] Jansen JG (2006). Strand-biased defect in C/G transversions in hypermutating immunoglobulin genes in Rev1-deficient mice. J Exp Med.

[66] Clapcote, S. J. & Roder, J. C. Simplex PCR assay for sex determination in mice. *Biotechnique*s **38** 702, 704, 706 (2005). 10.2144/05385BM05 15945368

[67] Schnaitman C, Greenawalt JW (1968). Enzymatic properties of the inner and outer membranes of rat liver mitochondria. J. Cell Biol..

[68] Spinazzi M, Casarin A, Pertegato V, Salviati L, Angelini C (2012). Assessment of mitochondrial respiratory chain enzymatic activities on tissues and cultured cells. Nat. Protoc..

[69] Venegas, V., Wang, J., Dimmock, D. & Wong, L.-J. Real-time quantitative PCR analysis of mitochondrial DNA content. *Curr. Protoc. Hum. Genet*. Chapter 19, Unit19.7 (2011). 10.1002/0471142905.hg1907s68 21234878

[70] Dingley S, Chapman KA, Falk MJ (2012). Fluorescence-activated cell sorting analysis of mitochondrial content, membrane potential, and matrix oxidant burden in human lymphoblastoid cell lines. Methods Mol. Biol..

[71] Klionsky DJ (2016). Guidelines for the use and interpretation of assays for monitoring autophagy (3rd edition. Autophagy.

[72] Bergersen LH, Storm-Mathisen J, Gundersen V (2008). Immunogold quantification of amino acids and proteins in complex subcellular compartments. Nat. Protoc..

