# Author Correction: Rev1 contributes to proper mitochondrial function via the PARP-NAD^+^-SIRT1-PGC1α axis

**DOI:** 10.1038/s41598-018-20586-9

**Published:** 2018-03-06

**Authors:** Nima Borhan Fakouri, Jon Ambæk Durhuus, Christine Elisabeth Regnell, Maria Angleys, Claus Desler, Md Mahdi Hasan-Olive, Ana Martín-Pardillos, Anastasia Tsaalbi-Shtylik, Kirsten Thomsen, Martin Lauritzen, Vilhelm A. Bohr, Niels de Wind, Linda Hildegard Bergersen, Lene Juel Rasmussen

**Affiliations:** 1 Center for Healthy Aging, Department of Cellular and Molecular Medicine, University of Copenhagen, Copenhagen, Denmark; 2 Department of Oral Biology, University of Oslo, Oslo, Norway; 3 Leiden University Medical Center, Leiden, Netherlands; 4 Center for Healthy Aging, Department of Neuroscience and Pharmacology, University of Copenhagen, Copenhagen, Denmark; 5 Department of Clinical Neurophysiology, Rigshospitalet, 2600 Glostrup Denmark; 6 National Institute on Aging, NIH, Baltimore, USA

Correction to: *Scientific Reports* 10.1038/s41598-017-12662-3, published online 02 October 2017

The original version of this article contained an error in the spelling of the author Md Mahdi Hasan-Olive, which was incorrectly given as Mahdi Hasan- Olive.

This error has now been corrected in the PDF and HTML versions of the paper.

