# Supplementary material for: Rev1 contributes to proper mitochondrial function via the PARP-NAD+-SIRT1-PGC1α axis
Source: Sci Rep. 2017 Oct 2;7:12480. doi: 10.1038/s41598-017-12662-3 (PMC5624938; doi:10.1038/s41598-017-12662-3)
Supplement: Supplementary file 1 — Supplementary Dataset 1 [file 41598_2017_12662_MOESM1_ESM.doc]

**Rev1 contributes to proper mitochondrial function via the PARP-NAD^+^-SIRT1-PGC1α axis**

Nima Borhan Fakouri^1^, Jon Ambæk Durhuus^1^, Christine Elisabeth Regnell^2,4^, Maria Angleys^1^, Claus Desler^1^, MD Mahdi Hasan- Olive^2^, Ana Martín-Pardillos^3^, Anastasia Tsaalbi-Shtylik^3^, Kirsten Thomsen^4^, Martin Lauritzen^4,5^, Vilhelm A. Bohr^1,6^, Niels de Wind^3^, Linda Hildegard Bergersen^2,4^, Lene Juel Rasmussen^1*^

^1^ Center for Healthy Aging, Department of Cellular and Molecular Medicine, University of Copenhagen, Denmark

^2^ Department of Oral Biology, University of Oslo, Norway

^3^ Leiden University Medical Center, Leiden, Netherlands

^4^ Center for Healthy Aging, Department of Neuroscience and Pharmacology, University of Copenhagen, Denmark

^5^ Department of Clinical Neurophysiology, Rigshospitalet, 2600 Glostrup, Denmark

**^6^** National Institute on Aging, NIH, Baltimore, USA

* Corresponding author: Lene Juel Rasmussen, lenera@sund.ku.dk

**
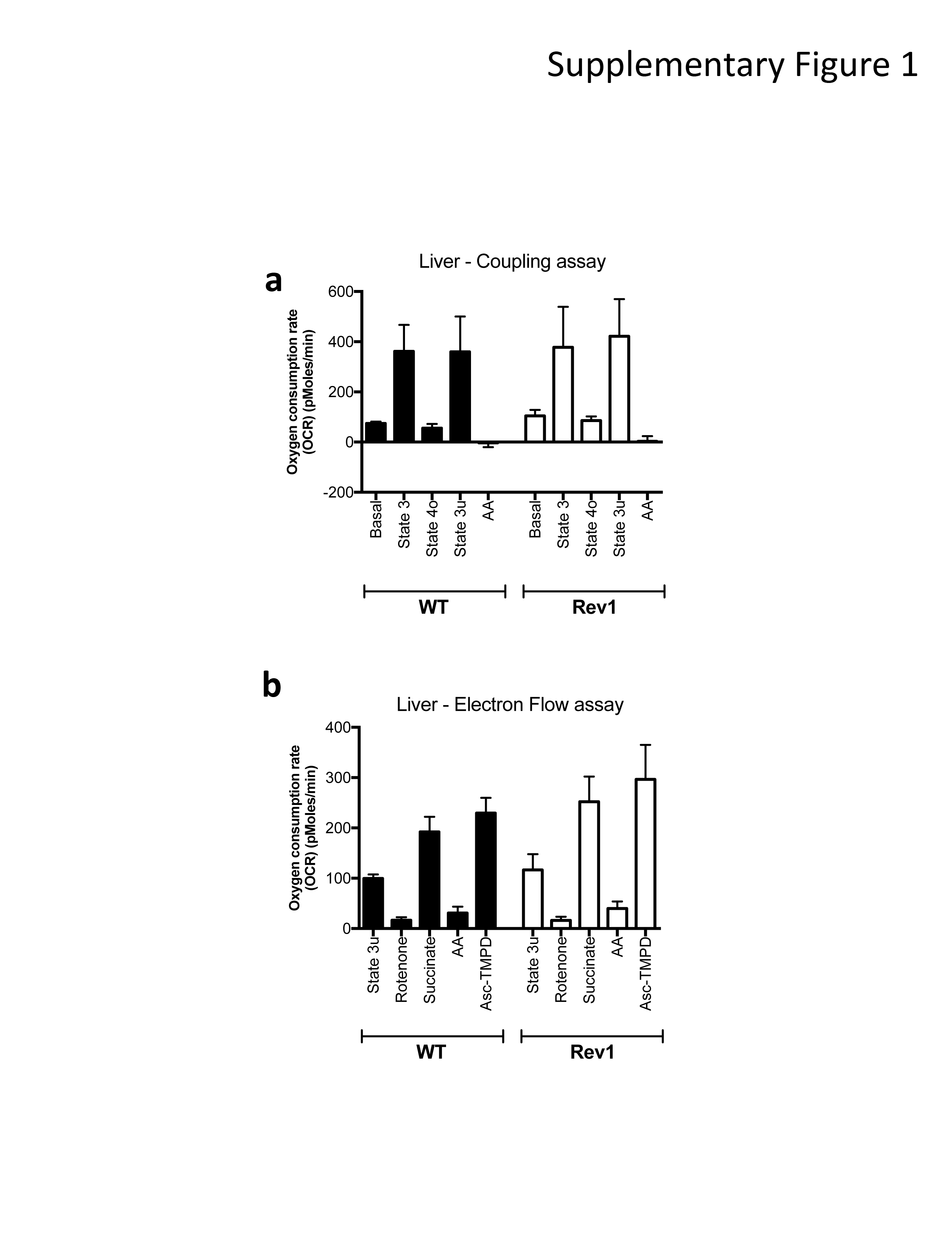
**

**Supplementary Figure 1. Analysis of mitochondrial function in hepatocytes of 5 months old WT and *Rev1^–/–^*** **mice. a and b)** Analysis of electron flow and coupling assay of the isolated mitochondria from the hepatocytes. Coupling assay: State 3 is initiated by addition of ADP, state 4o induced with addition of oligomycin, state 3u initiated by addition of FCCP and the assay was terminated by addition antimycin A (AA). The coupling assay was initiated by addition of FCCP followed by injection of rotenone and succinate, followed by AA and tetramethyl-p-phenylenediamine (TMPD).


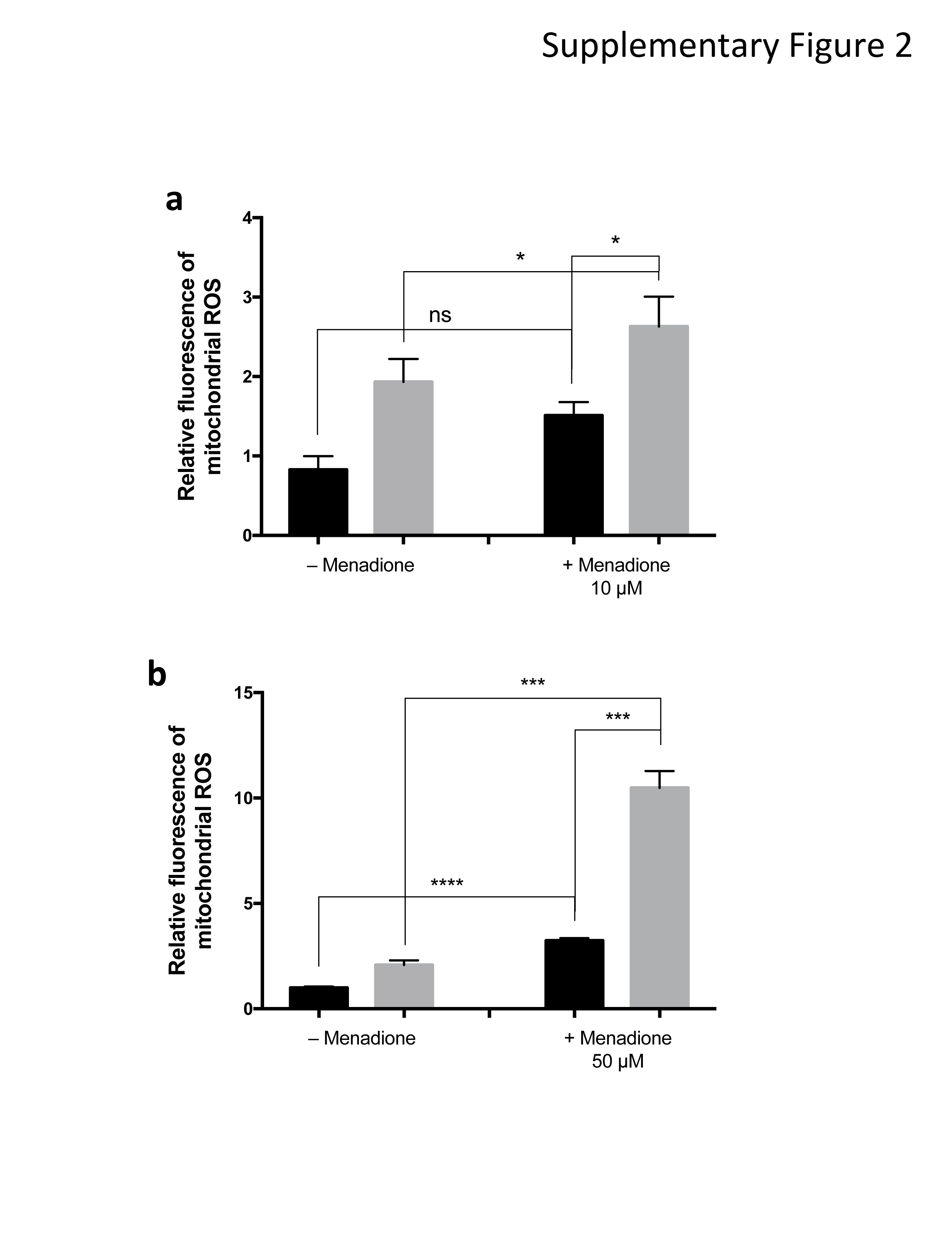


**Supplementary Figure 2. Assessment of mitochondrial ROS (mtROS) after treatment with menadione using MitoSOX Red and flowcytometry.** Cells cultured for 3 weeks prior to the measurement of mtROS. Cells treated with 10 µM menadione (a) and 50 µM (b) for 1hr prior to analysis by flowcytometry. The control group from each genotype was left untreated (n=3; df=2).

n= sample number; t=t value; df= degree of freedom; *p < 0.05. **p <0.01. ***p <0.001. ****p <0.0001. Data presented are mean ± M.S.E.

**
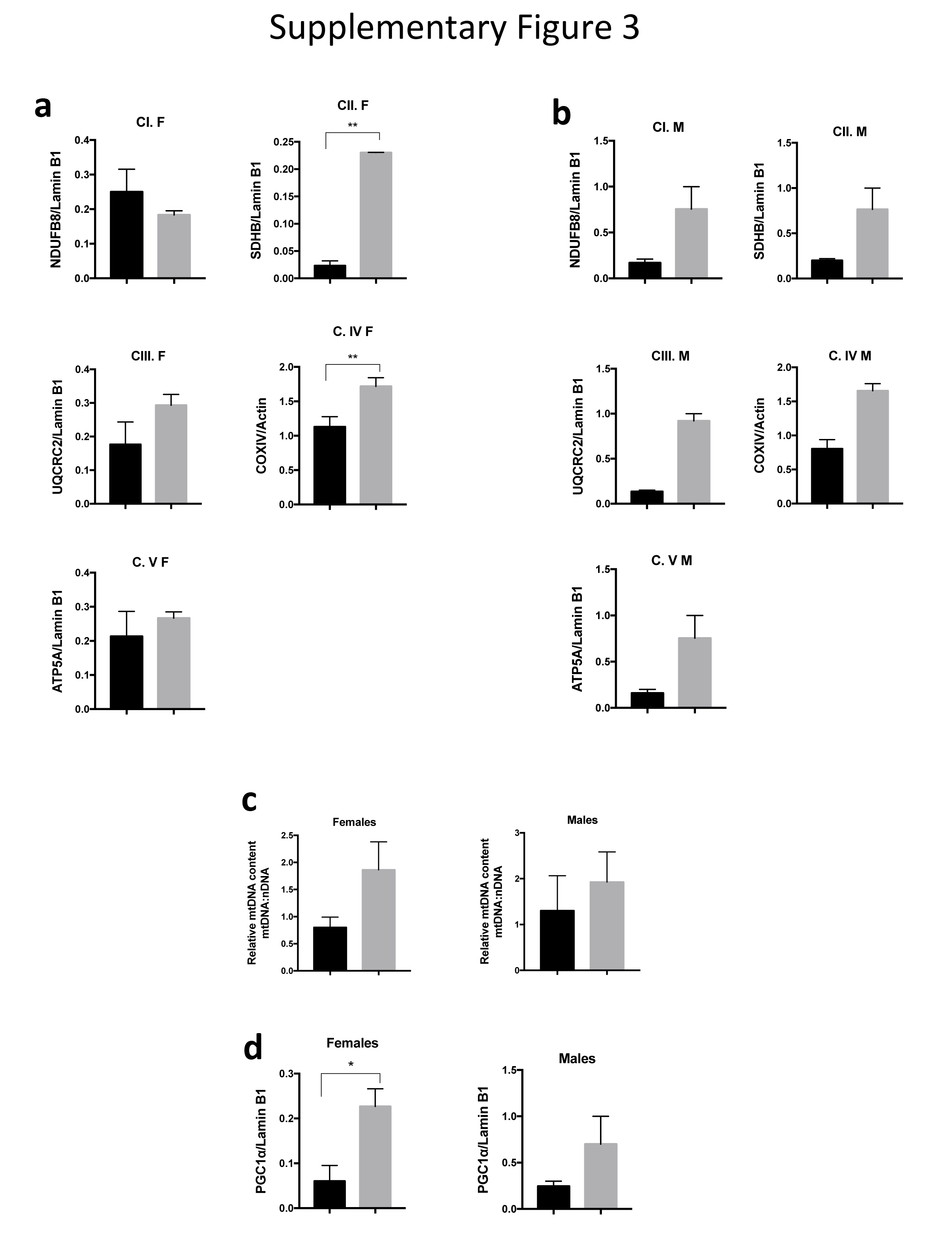
**

**Supplementary Figure 3. A and B)** Immunoblot analysis of the subunits of mitochondrial complexes in liver according to the gender of the mice: Females (a) males (b). C. I (NDUFB8, NADH dehydrogenase 1 beta subcomplex subunit 8); C. II: SDHB (Succinate dehydrogenase iron-sulfur subunit); C. III: UQCRC2 (Cytochrome b-c1 complex subunit 2); C. IV: MTCO1 (Cytochrome c oxidase subunit 1); C. V: ATP5A (ATP synthase subunit alpha). **c)** Analysis of mtDNA content by qPCR in females and males. The mtDNA content was calculated as the mtDNA/nDNA ratio using primers specific for the mitochondrial TrnL1and nuclear gene, β-2-microglobulin (β2M). **d)** Quantification of PGC1α protein level in the liver of WT and *Rev1^–/–^* 5 months old mice according to their gender. n= sample number; t=t value; df= degree of freedom; *p < 0.05. **p <0.01. ***p <0.001. ****p <0.0001. Data presented are mean ± M.S.E.

**
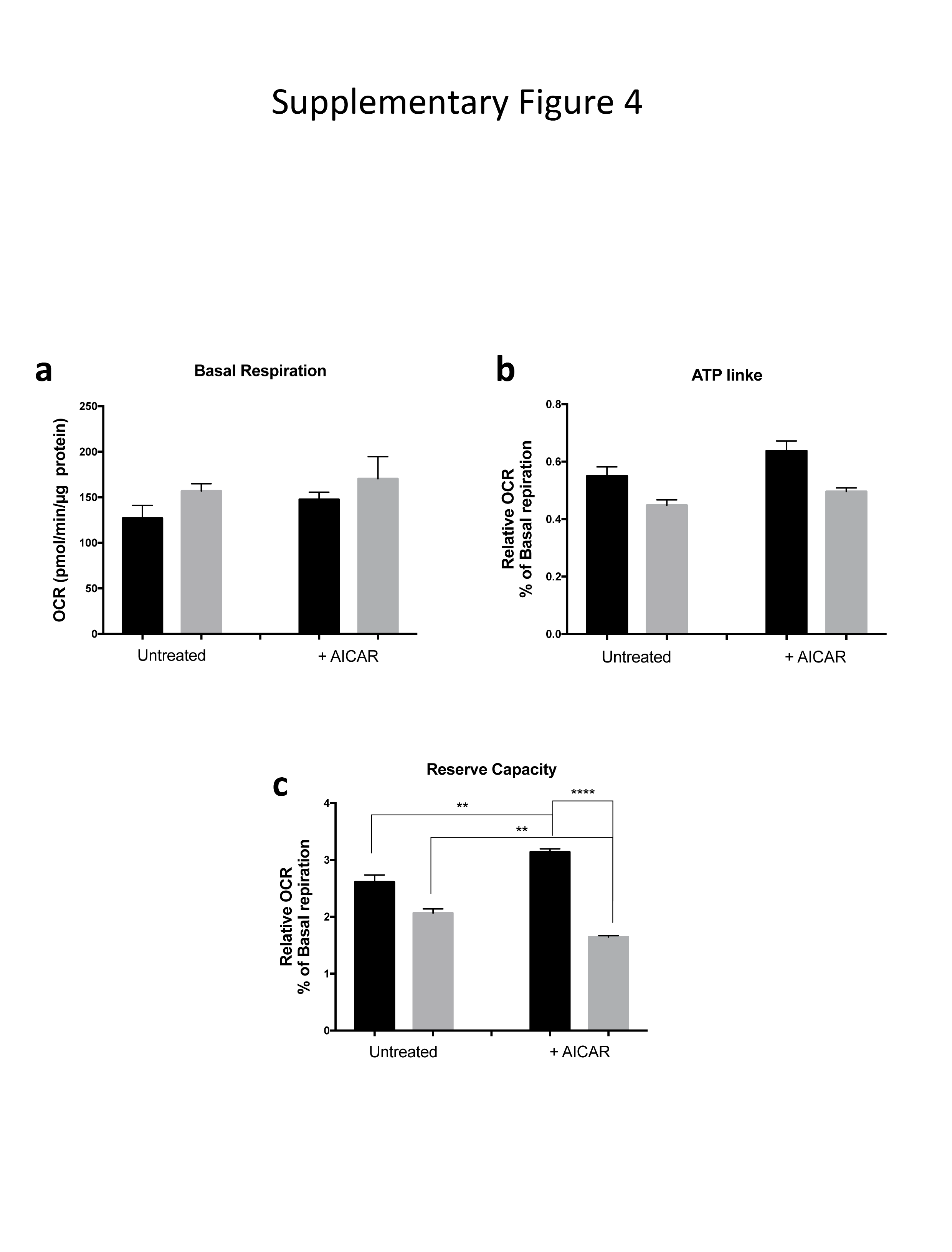
**

**Supplementary Figure 4. Mitochondrial bioenergetics in WT and *Rev1^–/–^* MEFs untreated and treated with AICAR.** Cells treated with 0.25 mM AICAR for 96 hrs prior to the assessment of mitochondrial bioenergetics. **a)** Basal respiration after normalizing to total protein content. **b)** ATP linked respiration and reserve capacity between WT and *Rev1^–/–^*, untreated and treated. ATP linked respiration and reserve capacity are represented as relative decrease and increase in OCR to basal respiration respectively. n= sample number; t=t value; df= degree of freedom; *p < 0.05. **p <0.01. ***p <0.001. ****p <0.0001. Data presented are mean ± M.S.E.


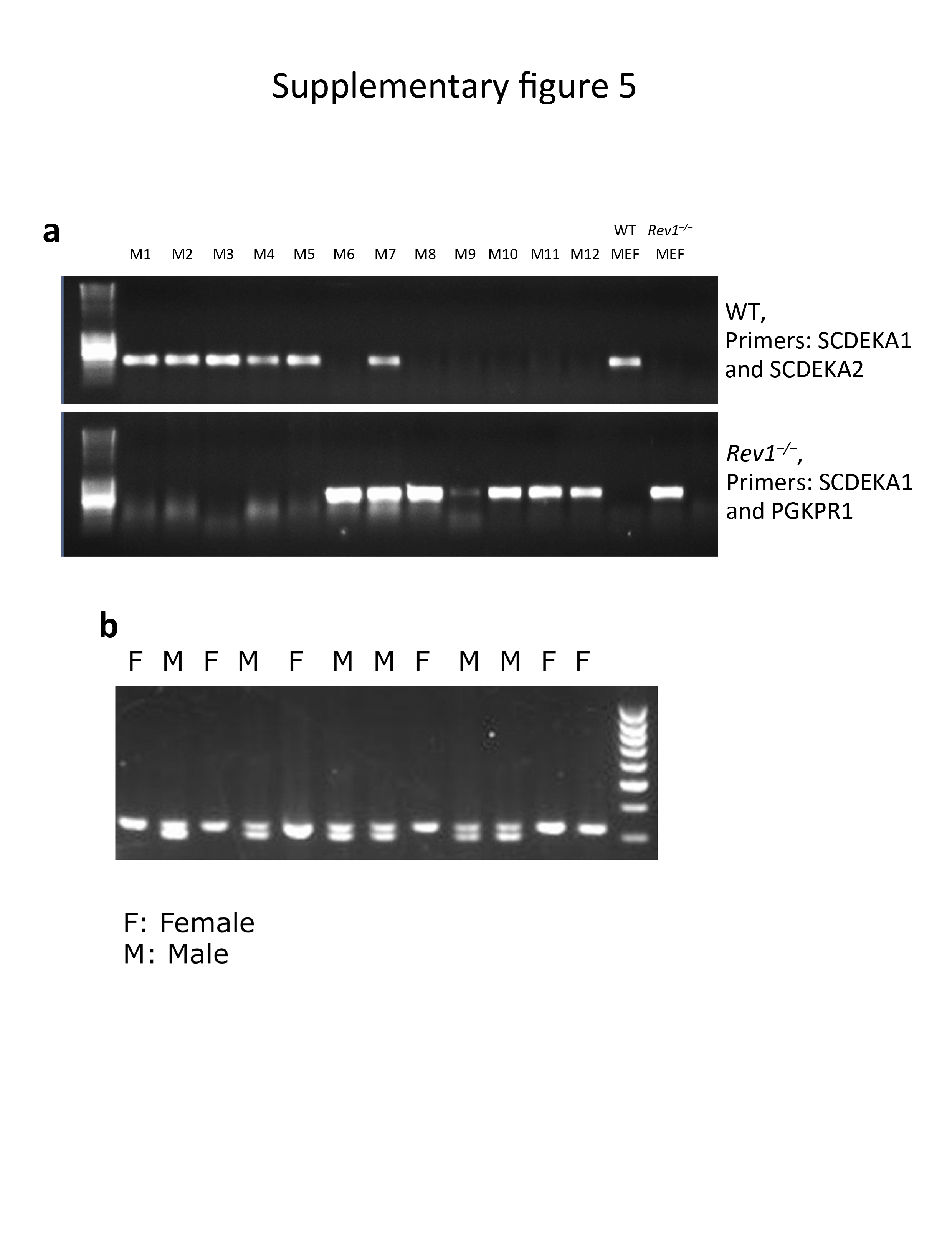


**Supplementary Figure 5. Genotyping and Sex determination in mice.** The genotype of the mice (liver tissue) and MEFs were determined according to Jansen et al., 2006 ^59^ using PCR reaction as described (35 cycles at 94°C for 60 s, 53°C for 60 s, and 72°C for 90 s) (a). The PCR products were loaded on a TBE agarose gel, stained with ethidium bromide and visualized with BioRAD GelDoc™ XR Molecular Imager (BioRAD). Primers SCDEKA1 (5’-ATTGTGAGTCTCTAGCGTTTG -3) and SCDEKA2 (5’-GCTGGAATTGAAATTCTAGG -3’) amplifying the wild type allele, and primers SCDEKA1 and PGKPR1 (5’-GCTTCCATTGCTCAGCGGTG -3’) amplifying the mutant allele (M=mouse).

Sex determination in mice and MEF were performed by PCR according to (Clapcote SJ, Roder JC. [Simplex PCR assay for sex determination in mice.](https://www.ncbi.nlm.nih.gov/pubmed/15945368) Biotechniques. 2005 May;38(5):702, 704, 706. PMID: 15945368), using a single pair of primers (forward primer: CTGAAGCTTTTGGCTTTGAG, Reverse primer: CCACTGCCAAATTCTTTGG) that amplifies both a 302 bp X-chromosome product and a 331 bp Y-chromosome product. Reactions were measured on a Biometra thermocycler (Thermo Fisher Scientific) and incubated at 95°C for 5 min, followed by 35 cycles of 95°C for 20 s, 54°C for 1 min, and 72°C for 40 s, followed by 72°C for 10 min. The PCR products were loaded on a TBE agarose gel, stained with ethidium bromide and visualized with myECL imager (Thermo Fischer Scientific).


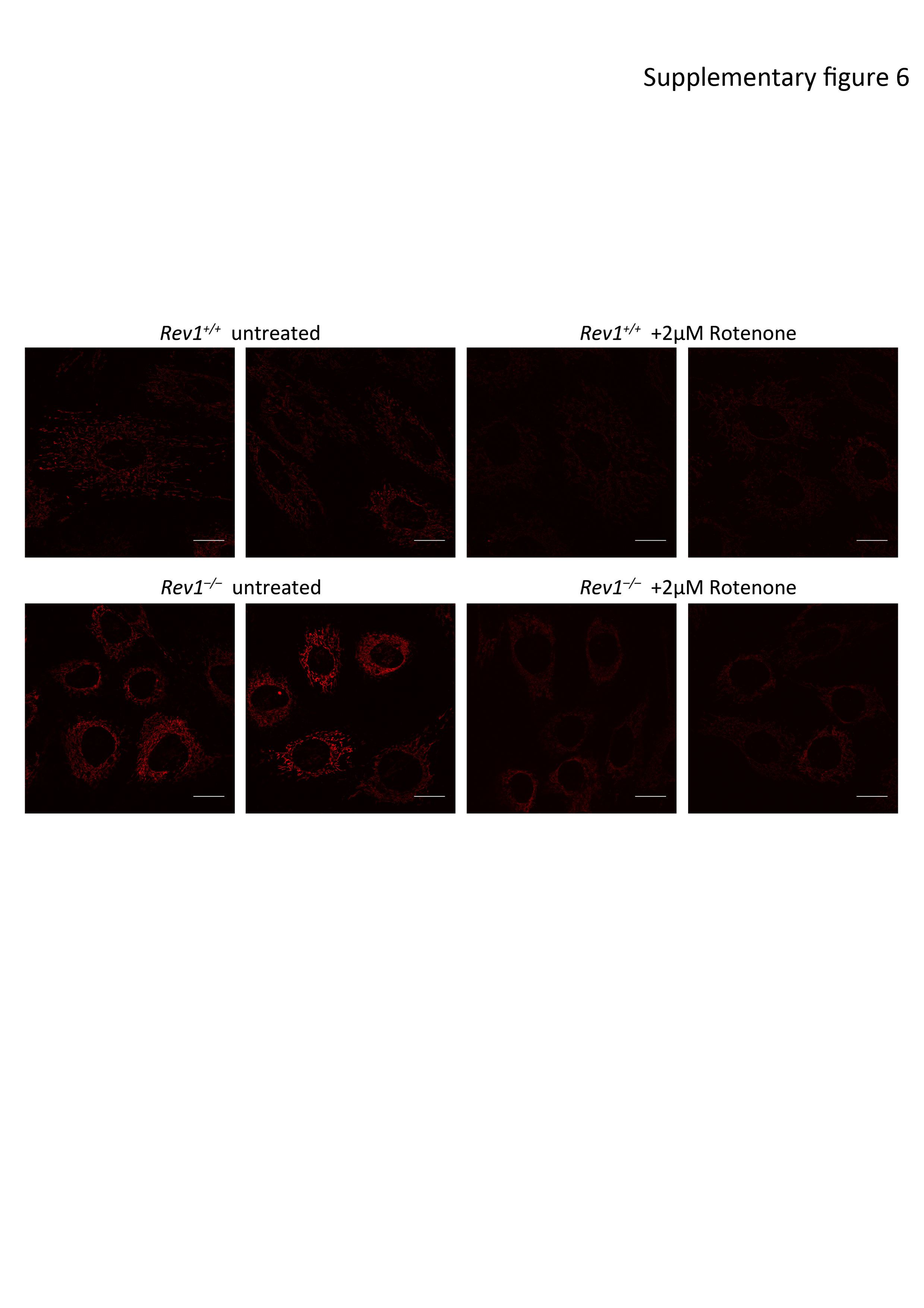


**Supplementary figure 6, Live cell imaging of mitochondrial membrane potential.** Treated *Rev1^+/+^* and *Rev1^–/–^* MEF cells incubated with complete medium containing 2µM rotenone for 5h prior to staining and live cell imaging (scale bar size = 20µm).


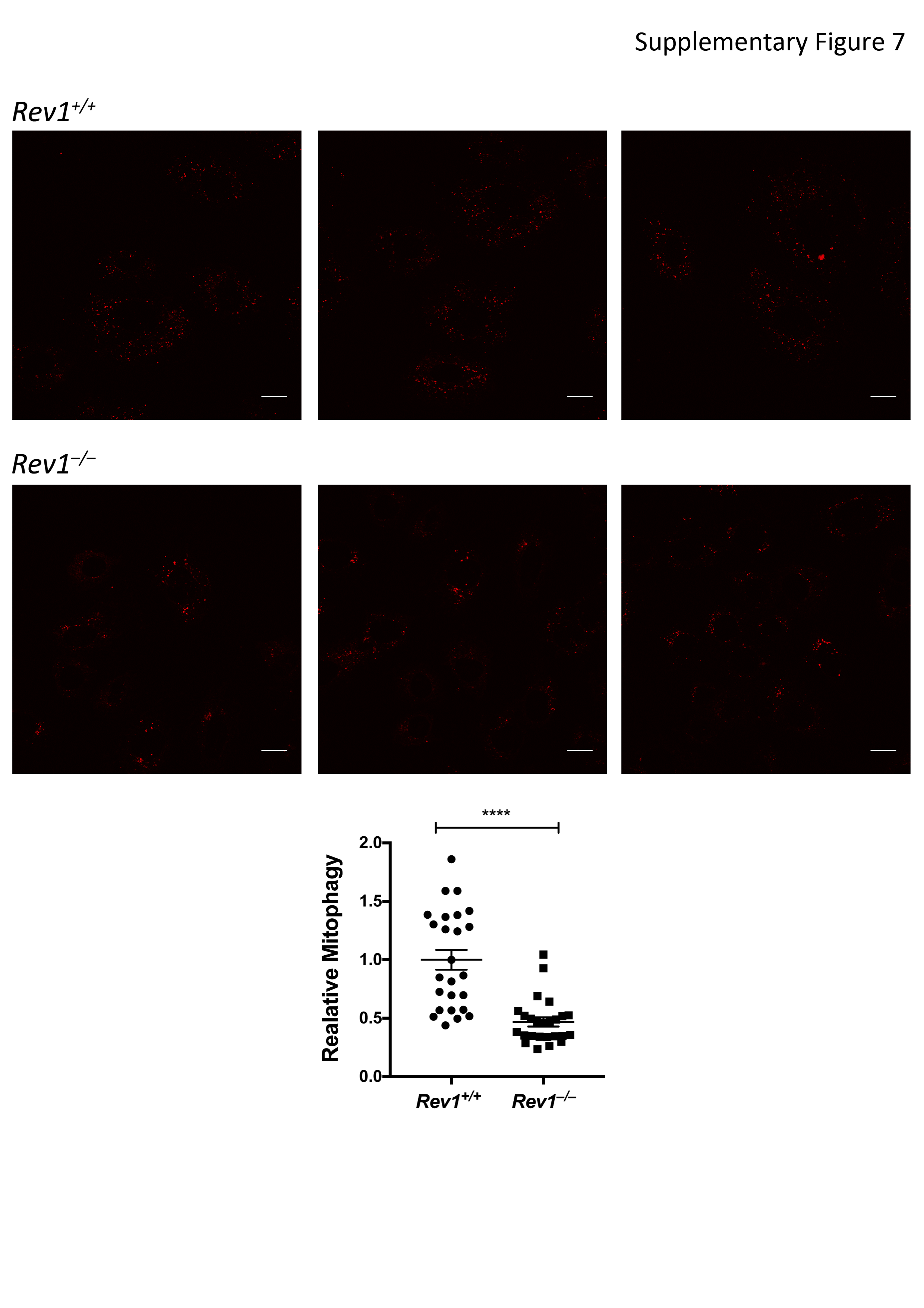


**Supplementary figure 7, Analysis of mitophagy in MEF cells by live cell imaging.** Mitophagy is analyzed by incubating *Rev1^+/+^* and *Rev1^–/–^* MEF cells with Dojindo mitophagy dye according to manufacturer procedures. Mitophagy is induced by treating the cells with 2µM rotenone for 5h prior to live cell imaging (scale bar = 20µm) (*P < 0.0001*; n=25).

**
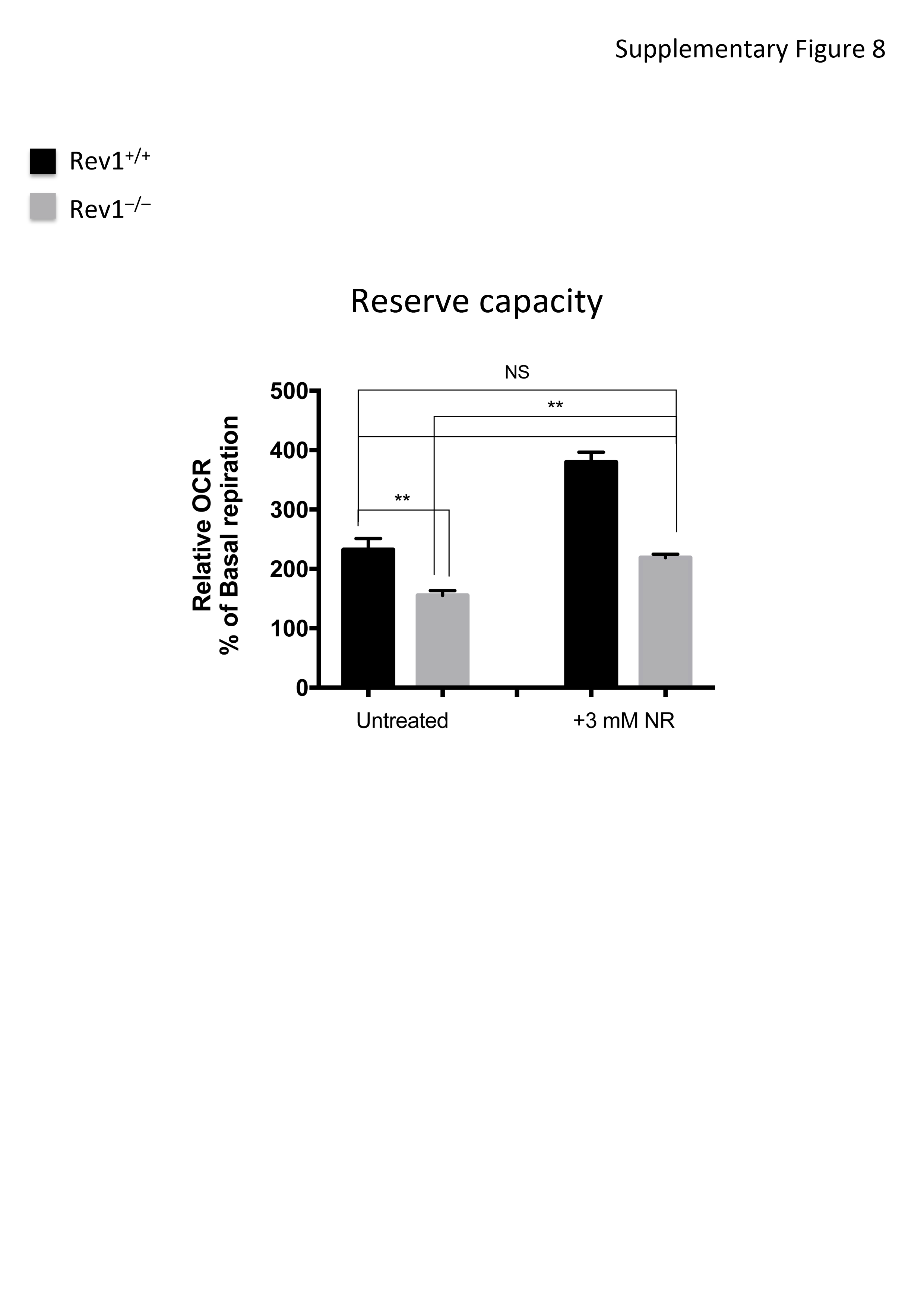
**

**Supplementary figure 8, Effect of Nicotinamide riboside (NR), a NAD^+^ precursor, supplementation on the reserve capacity of *Rev1^–/–^* and *Rev1^+/+^* MEF cells.**

Treatment with 3 mM NR for 72h enhance the mitochondrial function in of *Rev1^–/–^* MEFS as it increases the reserve capacity to the level of *Rev1^+/+^* MEF cells (n=3).


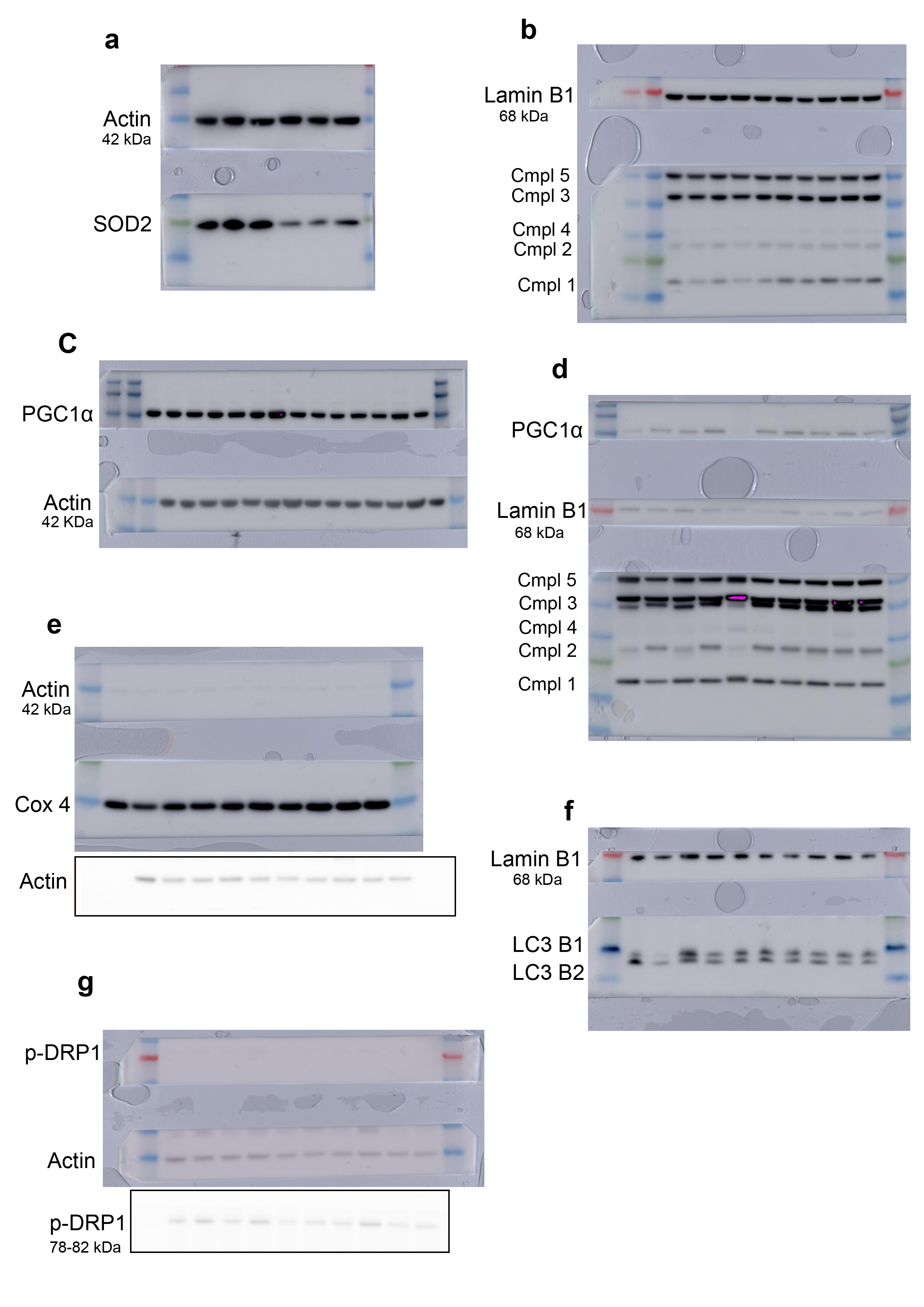


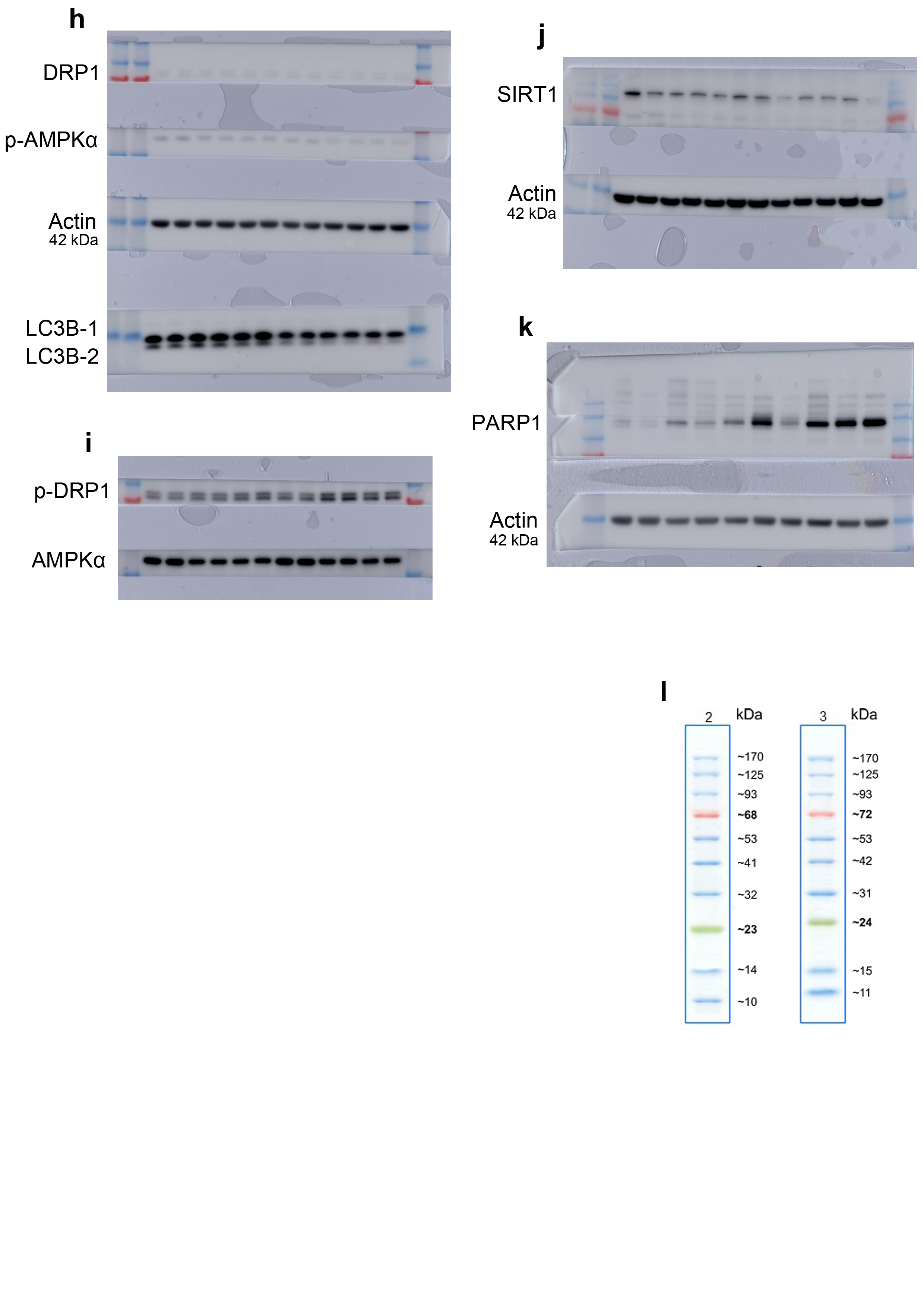


**Supplementary figure 9, Unprocessed western blot figures of membranes that are exposed initially for 1 minute using Amersham™ Imager 600.** After one minute exposure, the incremental mode is used to expose the membrane for different intervals and pictures were taken after every 10s, 20s, 30s, or 1 minute depending on the image that is obtained after primary 1 minute exposure. The best images were chose for processing and analysis using image J according to the guidelines. e, figure 1; b, c, d, e, figure 2, e: the boxed figure shows the unprocessed picture of longer exposure of membrane that contains actin at incremental mode. f, g, h, i, figure 4, g the boxed figure shows the unprocessed picture longer exposure of membrane that contains phospho-DRP1 at incremental mode. j and k figure 5. l, The picture of the abcam Prism Ultra Protein Ladder (10 - 180 kDa) (ab116027).
